# Optimal parameters of roadway supports for water prevention based on the FFA algorithm

**DOI:** 10.1371/journal.pone.0357258

**Published:** 2026-10-01

**Authors:** Chao Xu

**Affiliations:** School of Resources and Geosciences, China University of Mining and Technology, Xuzhou, China; Henan Polytechnic University, CHINA

## Abstract

Roadways are important paths in mining engineering. If the stability of roadway supports cannot be guaranteed, workers’ lives will be threatened. Therefore, the firefly algorithm (FFA) and FLAC3D numerical simulation were combined. Taking the roadway of the Banji Coal Mine as an example, the FFA was used to obtain the optimal grouting area and cable distribution design. This support scheme was then applied to engineering practice. The numerical simulation results were in good agreement with those of engineering practice, indicating that the proposed method is reliable and feasible and can be applied to determine the optimal design for supporting roadways in engineering practice.

## 1. Introduction

With the continuous deepening of coal mines and the expansion of the scope of roadway excavation, water inrush disasters have become among the most serious geological hazards, as they restrict safe mining, threaten the lives of underground workers and cause considerable economic losses for mining enterprises [1–5]. Roadway supports are the core measure used to control surrounding rock deformation and prevent water inrush, and the support parameters directly affect the stability of the rock surrounding roadways as well as water prevention and control. Reasonable optimal designs of roadway support parameters can not only improve the antideformation ability of the surrounding rock and reduce the occurrence probability of water inrush accidents [6–8] but also save support materials and reduce engineering costs, which has important theoretical significance and engineering application value for realizing safe, efficient and economical mining [9–13].

To determine the optimal roadway support scheme that can effectively ensure the stability of rock surrounding roadways, resist water damage, and meet economic engineering requirements, a series of systematic and in-depth experiments were conducted. These experiments focused mainly on understanding the complex fluid‒mechanical coupling effects in rock masses surrounding roadways under different geological conditions (such as different water-bearing strata, fractured rock masses, and burial depths) [14,15]. During the experimental process, various parameters, including rock mass permeability, stress distribution, deformation characteristics, and water seepage, were monitored in real time. On the basis of a comprehensive analysis of experimental data, corresponding water prevention measures and roadway support schemes were proposed from the perspective of controlling engineering costs to ensure that the proposed schemes not only have good stability and water resistance but also avoid excessive economic investment in engineering [16].

With respect to numerical simulation, which is an important auxiliary means for designing roadway supports, researchers usually propose a variety of alternative roadway support schemes first. These schemes differ in terms of the key support parameters, such as the type of support material (e.g., anchor rod, anchor cable, or shotcrete), support spacing, support strength, and the layout of the support structure. The FLAC3D numerical simulation software (a professional geotechnical engineering simulation tool) is subsequently used to simulate the stress‒strain state, deformation law, and stability of the rock surrounding roadways in each support scheme. Finally, on the basis of the numerical simulation results (such as the degree of roadway convergence, surrounding rock stress concentration coefficient, and support structure force), the optimal roadway support scheme is selected from the limited number of support design schemes through comparative analysis [17–19]. While, many other scholars try to obtain the optimal support from the perspective of stress and strain, such as, Li et al. [20] performed the field measurement and numerical simulation, the results reveal asymmetric stress and principal stress axis deflection in mining-damaged roadways. An integrated roof cutting and asymmetric support technology is proposed, whose effectiveness is verified via field monitoring.

Meanwhile, to investigate the influence of fault geometry and roadway cross-section shape on the collapse of roadways, some experiments were conducted, and the corresponding results were obtained. Wang et al. [21] conducted the uniaxial compression tests on double-hole specimens with pre-embedded joints were conducte, with DIC and AE used to monitor strain evolution and crack development. It can be found that circular twin roadways are more stable than square ones. Collapse stress drops to the minimum at α = 45°before rising as α increases from 0°to 90°. Numerical analysis supplements the tests, offering guidance for roadway construction in jointed rock masses. Mei et al. [22] developed a theoretical-numerical framework for transient P- and SV-wave responses of twin arbitrary-section tunnels via conformal mapping and complex variable theory, validated by limit cases. CASRock simulations further reveal static-dynamic coupled elastoplastic failure, offering references for deep multi-tunnel support design. In Yan’s studies [23], model tests and validated numerical simulations are adopted to explore structural mechanical responses, surrounding rock earth pressure and existing lining safety. Parametric analysis shows rising buffer stiffness increases lining internal force, and a 10 MPa elastic modulus buffer is recommended. The optimized scheme adapts to 1.46–3.06 m ultra-small clearance and cuts concrete consumption, providing references for mitigating construction disturbance to adjacent tunnels. These studies provide novel insights for research on roadway stability.

However, through the systematic analysis and induction of the research results of existing scholars, there are obvious limitations in the process used to determine the optimal roadway support scheme in most previous studies. Specifically, the process of obtaining the optimal support scheme is not scientific or rigorous enough. Owing to the lack of a unified and objective evaluation standard and quantitative analysis method, the process of selecting the support scheme and the final optimal support design determined are influenced strongly by the subjective experience and professional judgement of researchers. This subjectivity often leads to deviations in the optimal support scheme, making it unable to fully adapt to the actual geological conditions of the roadway and thus affecting the stability of the roadway support and the rationality of the investment.

To overcome the above limitations of existing studies and improve the scientific rigor, objectivity, and reliability of determining the optimal roadway support scheme, this study combines the firefly algorithm (FFA) with FLAC3D numerical simulation to establish a new method for determining the optimal roadway support design. In this method, the firefly algorithm, which has the advantages of strong global search ability, fast convergence, and easy implementation, is used as the core optimization method to dynamically update and optimize the key support parameters (such as the anchor rod length, anchor cable spacing, and grouting area). To verify the effectiveness and practicality of the proposed method, the optimized roadway support design scheme was applied to actual engineering practice. During the engineering implementation process, key monitoring indicators, such as the deformation of the rock surrounding roadways and water seepage volume, were recorded in detail. A comparison of the actual monitoring data with the numerical simulation results and the design requirements reveals that the deformation of the rock surrounding roadways with the optimized support scheme is within the allowable range, the support structure is in a stable stress state, and there is no obvious water seepage. These results indicate that the proposed method combining the FFA and FLAC3D numerical simulation is reliable and workable and can provide an effective technical reference for the optimal design of roadway supports under complex geological conditions.

## 2. Determining the optimal roadway support by using firefly algorithm

### 2.1. Firefly algorithm

The firefly algorithm (FFA) is a swarm intelligence optimization algorithm inspired by the flashing behaviour of fireflies that Xin-She Yang proposed in 2010 [24]. Its core logic is that fireflies move towards brighter counterparts, with brightness tied to the objective function value and attractiveness decaying with distance. The FFA has several advantages: first, it features a simple and intuitive mechanism with few adjustable parameters, making it easy to understand, implement and program. Second, it exhibits strong global search capability, especially for multimodal optimization problems, as its attraction mechanism allows fireflies to automatically cluster into subgroups and effectively avoid premature convergence to local optima. Third, it achieves a good balance between global exploration and local exploitation by adjusting randomness and absorption coefficients. The detailed process of FFA algorithm is described below.

Before the process is considered, it is critical to grasp the three foundational assumptions:

(1) All fireflies are unisex—any firefly will be attracted to all other brighter fireflies (no sex constraints).
(2) Attractiveness is proportional to brightness (brighter fireflies are more attractive) and decays exponentially with distance (attractiveness decreases as distance increases).
(3) The brightness of a firefly is determined by the objective function value: For maximization problems, the brightness I∝f(x) (higher f(x) = brighter). For minimization problems, the brightness I∝1/f(x) (lower f(x) = brighter).

The main procedure for the FFA is described as follows:

Step 1: Initialize the Algorithm Parameters

Define the core hyperparameters (adjustable based on the optimization problem). n: Number of fireflies (swarm size, typically 20–50). d: Dimension of the optimization problem (number of variables to optimize). α: Randomization parameter (step size, usually 0.01–1.0). $\beta_{\text{0}}$: Initial attractiveness at distance 0 (base attractiveness, usually 1.0). γ: Light absorption coefficient (controls attractiveness decay, usually 0.1–10). max_iter: Maximum number of iterations (stopping criterion). Search range: [$x_{min}$, $x_{max}$] for each dimension.

Step 2: Initialize the Firefly Population

Randomly generate the initial position of each firefly in the search space:


$$\begin{array}{c} X_{i}=(x_{i1},x_{i2},•••,x_{id}),i=1,2,•••,n \end{array}$$ (1)

where xij ~ is _uniform_([$x_{min}$, $x_{max}$) (uniform random distribution).

Step 3: Calculate the Brightness and Attractiveness

Brightness calculation: For minimization, $I_{i}=1/(1+f(X_{i}))$ (division is avoided by 0 if $f\left(X_{i}\right)$ =0). For maximization, $I_{i}=f\left(X_{i}\right)$. (Note: $f\left(X_{i}\right)$ is the objective function value of firefly i.) Distance between fireflies i and j: The Euclidean distance (most common) can be expressed as follows:


$$\begin{array}{c} r_{ij}=∥X_{i}-X_{j}∥=\sqrt{\sum_{k=1}^{d}(x_{ik}-x_{jk}{)}^{\text{2}}} \end{array}$$ (2)

Attractiveness calculation (decays with distance):


$$\begin{array}{c} \beta \left(r_{ij}\right)=\beta_{\text{0}}e^{-\gamma {r_{ij}}^{\text{2}}} \end{array}$$ (3)

Exponential decay ensures that attractiveness decreases rapidly with distance; γ controls the decay rate.

Step 4: Update the Firefly Positions

For each pair of fireflies i and j,

If $I_{j}>I_{i}$ (firefly j is brighter), firefly i moves towards j:


$$\begin{array}{c} X_{i}=X_{i}+\beta \left(r_{ij}\right)•(X_{j}-X_{i})+\alpha •(rand+0.5) \end{array}$$ (4)

$\beta \left(r_{ij}\right)•(X_{j}-X_{i})$: Attractive movement towards the brighter firefly. α•(rand+0.5): Random perturbation (exploration of the search space, rand∈[0,1]).

If $I_{j}<I_{i}$, Firefly i moves randomly (no attraction).

The brightest firefly (global optimal thus far) only moves randomly (to avoid local optimal values):


$$\begin{array}{c} X_{best}=X_{best}+\alpha •(rand-0.5) \end{array}$$ (5)

Step 5: Evaluate the New Positions

Recalculate the brightness $I_{i}$ for all fireflies with updated positions, and update the global best firefly (brightest one).

Step 6: Check the Termination Criterion

If the number of iterations reaches max_iter or if the objective function value converges (no significant change for multiple iterations), stop the algorithm.

Otherwise, return to Step 3 and repeat.

### 2.2. Theory of fluid‒mechanical coupling in FLAC3D

The fluid‒mechanical coupling analysis in FLAC3D is grounded in the quasistatic Biot’s consolidation theory, which elaborates the bidirectional interactive mechanism between the seepage of single-phase fluid in porous geomaterials and the deformation/failure of the solid skeleton [25,26]. The numerical solution adopts the explicit finite difference method to solve the governing equations of solid mechanics and fluid flow alternately and to calculate the dynamic coupling of pore pressure and the stress‒strain field.

FLAC3D adopts the staggered explicit solution scheme for FM coupling, which solves the solid mechanical equilibrium equation and the fluid seepage control equation alternately in each time step without solving the coupled equations simultaneously, thus reducing the computational complexity and improving the efficiency:

(1) Mechanical step: The displacement, strain and stress of the solid skeleton are calculated at the current pore pressure, and the volumetric strain $\varepsilon_{v}$ is updated;
(2) Hydraulic step: Based on the updated volumetric strain $\varepsilon_{v}$ and porosity φ, the fluid mass conservation equation is solved to update the pore pressure field p;
(3) Iteration: The above two steps are repeated until the preset calculation time or convergence condition is reached (e.g., the variation in pore pressure and displacement is less than the threshold).

The time step of the coupling calculation must satisfy the stability condition for both the mechanical and hydraulic fields: the mechanical time step $\Delta t_{m}$ is controlled by the wave velocity of the solid medium, and the hydraulic time step $\Delta t_{f}$ is controlled by the seepage velocity and grid size. FLAC3D automatically synchronizes or adjusts the two time steps to avoid numerical oscillation and ensure calculation stability.

Key Assumptions of FLAC3D FM Coupling Theory

(1) The fluid is single phase, and seepage conforms to Darcy’s law (laminar flow and low seepage velocity);
(2) The deformation of the solid skeleton is small (small strain assumption), and the large deformation can be considered by enabling large-strain mode;
(3) The porous medium is fully saturated (unsaturated seepage must be realized by the unsaturated fluid module with the van Genuchten model);
(4) The coupling is quasistatic, and the inertial effect of fluid flow is ignored (dynamic seepage can be considered in special settings);
(5) The solid particles and fluid are slightly compressible (an incompressible fluid can be set by assigning a large $K_{f}$).

### 2.3. Optimizing the roadway support

Before the numerical simulation model is constructed, the centre point of the roadway should be determined, which is O in Figs 1–3. Then, the area around centre point O is divided into 10 parts evenly by 10 lines, and the lengths of the ten lines are $l_{\text{1}},l_{\text{2}},•••,l_{10}$; on the basis of the coordinate $\left(x_{\text{0}},y_{\text{0}}\right)$ of the centre point O, the grouting area end point $p_{\text{1}},p_{\text{2}},•••,p_{10}$ can be obtained as follows:


$$\begin{array}{c} \left\{ \begin{array}{c} @l@x_{i}=x_{\text{0}}+l_{i}cos((i-1)•36)\\ y_{i}=y_{\text{0}}+l_{i}sin((i-1)•36) \end{array}\right.i=1,2,•••,10 \end{array}$$ (6)

Once ten end points $p_{\text{1}},p_{\text{2}},•••,p_{10}$ of the grouting area are determined, a closed line can be obtained, as shown by the red dotted line in Fig 1. The closed line is a smooth curve. The smooth curve is obtained on the basis of ten end points $p_{\text{1}},p_{\text{2}},•••,p_{10}$ and the cubic spline interpolation method.

**Fig 1:**
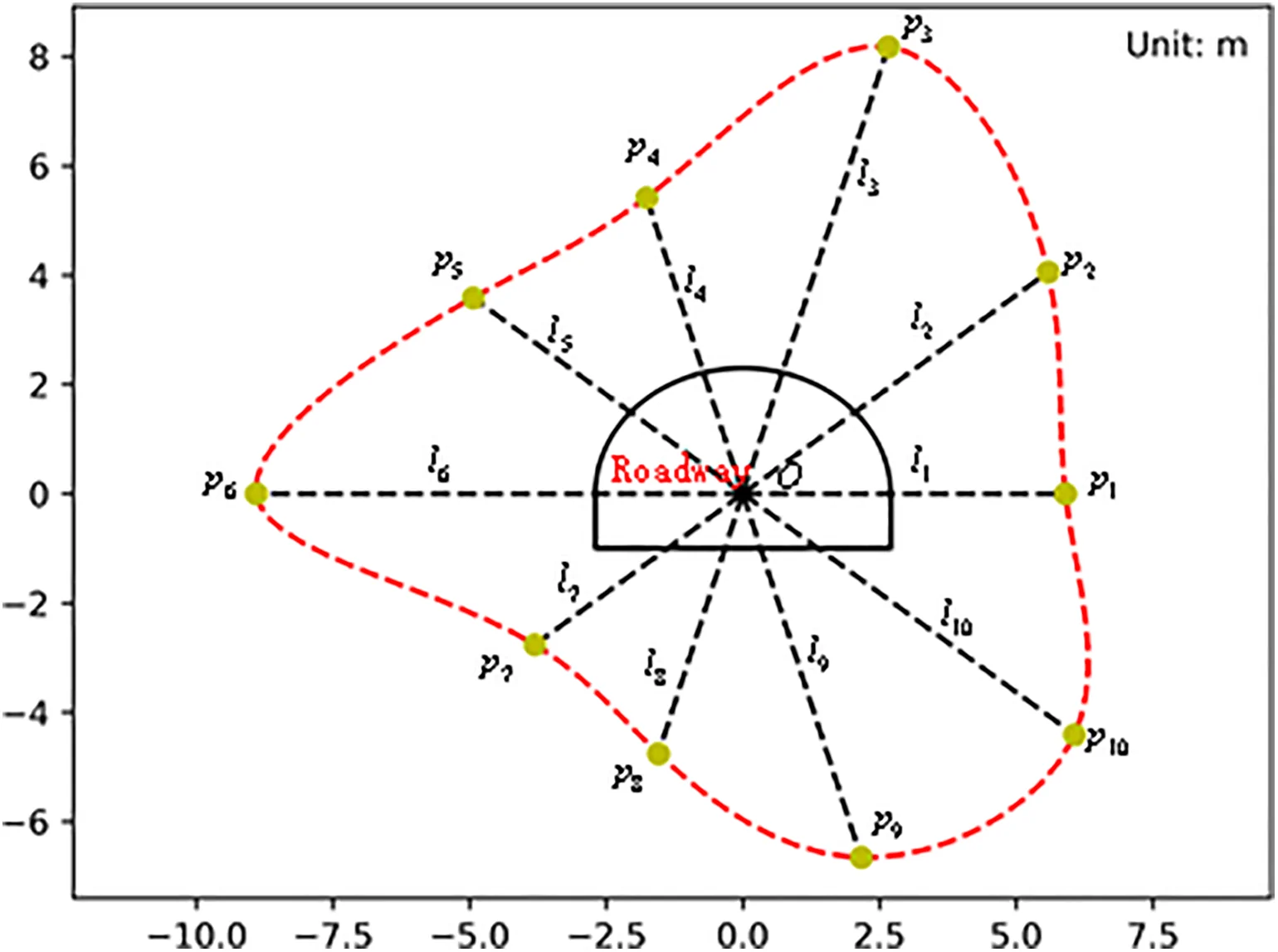
End point of the grouting area.

The area within the range of the closed line and the roadway edge is subsequently obtained, as shown in Fig 1. For the numerical simulation model, when the zone is within the range of the area, the zone is grouped and considered the grouting area, as shown in Fig 2 and Fig 3.

**Fig 2:**
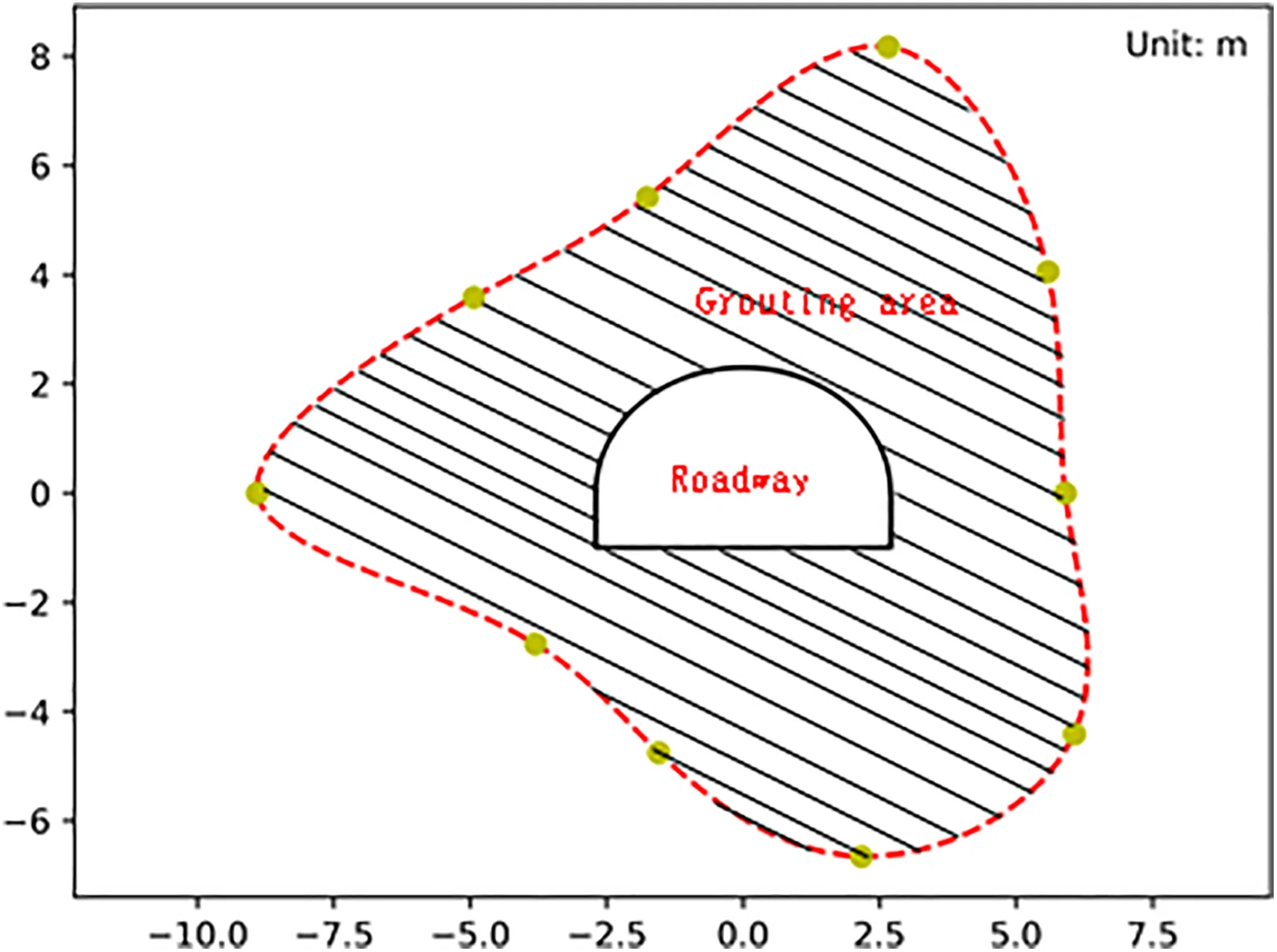
Grouting area determined on the basis of the roadway edge and the closed line.

**Fig 3:**
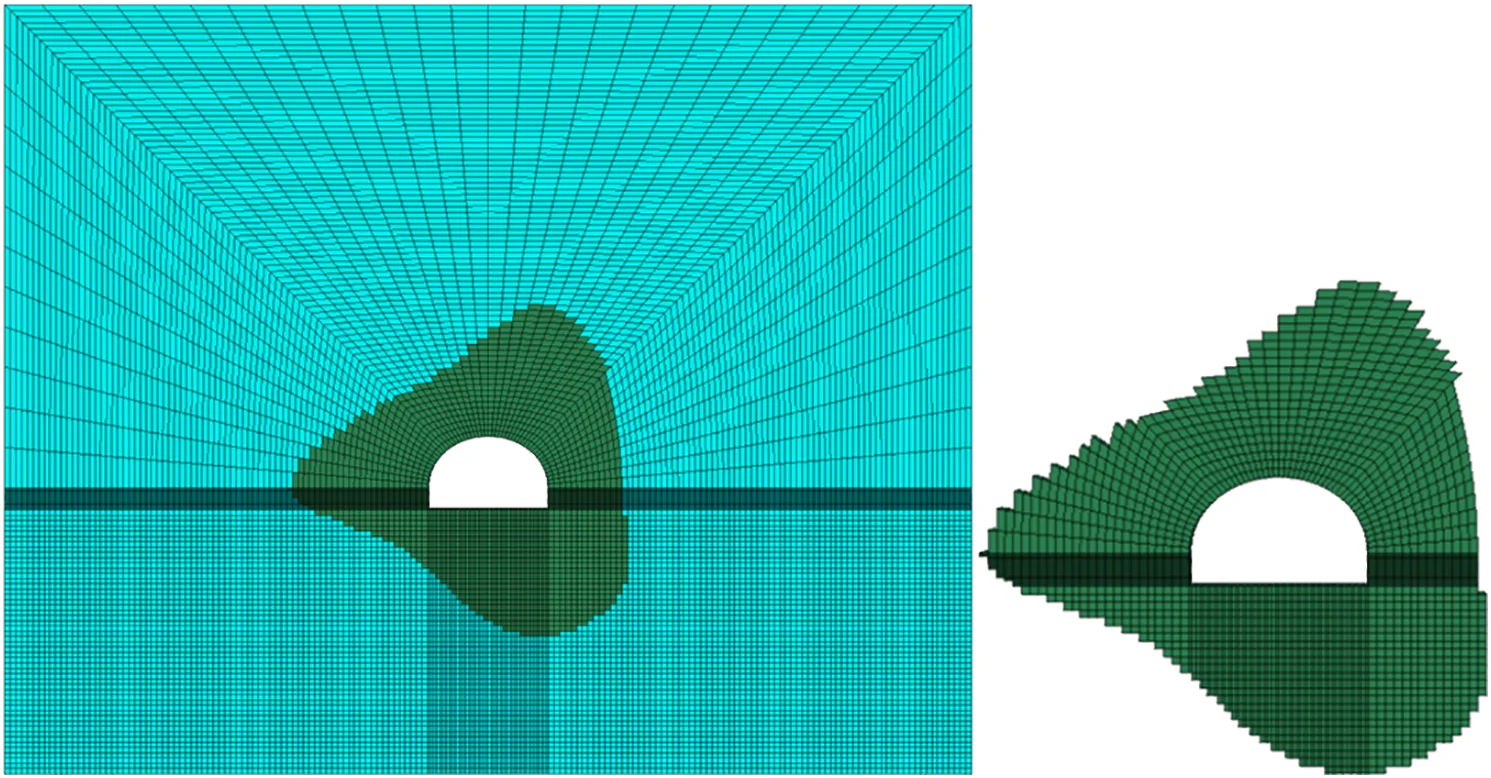
Grouting area in the numerical simulation model (FLAC3D).

Moreover, to reduce the deformation of the roadway, cables are used, and the cable distribution is shown in Figs 4–6.

**Fig 4:**
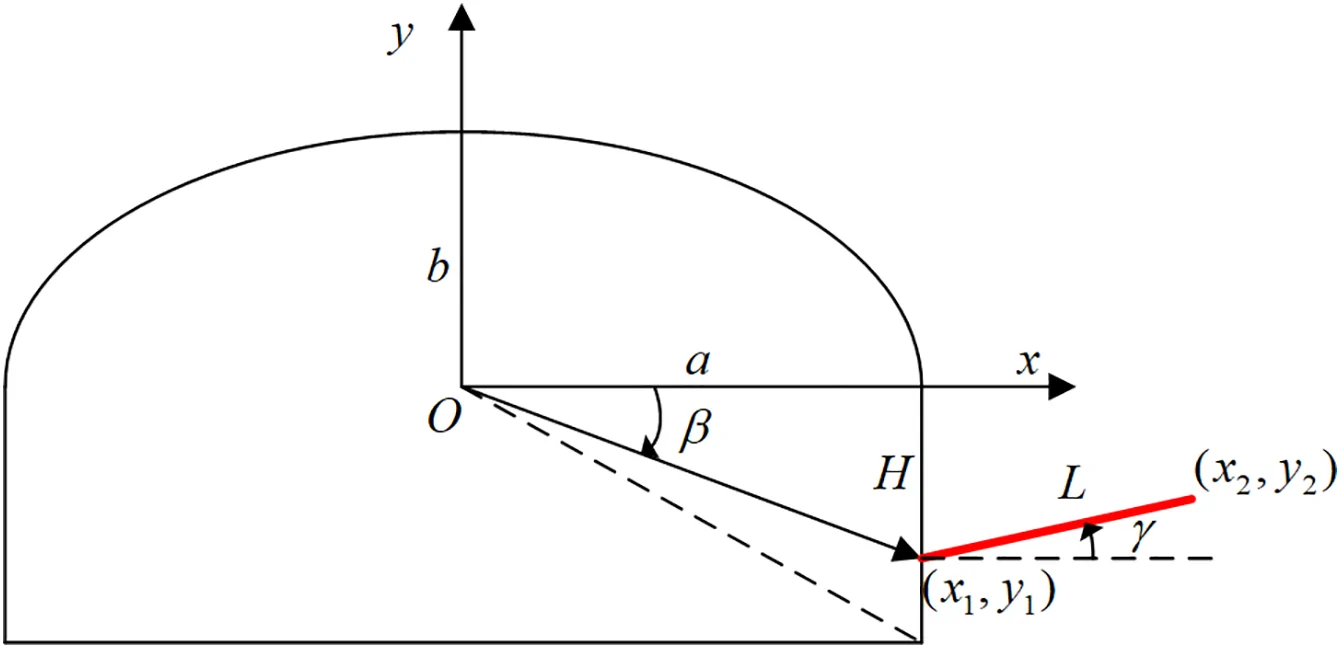
$-arctan\left(\frac{H}{a}\right)\leq \beta <0^{\circ }$.

**Fig 5:**
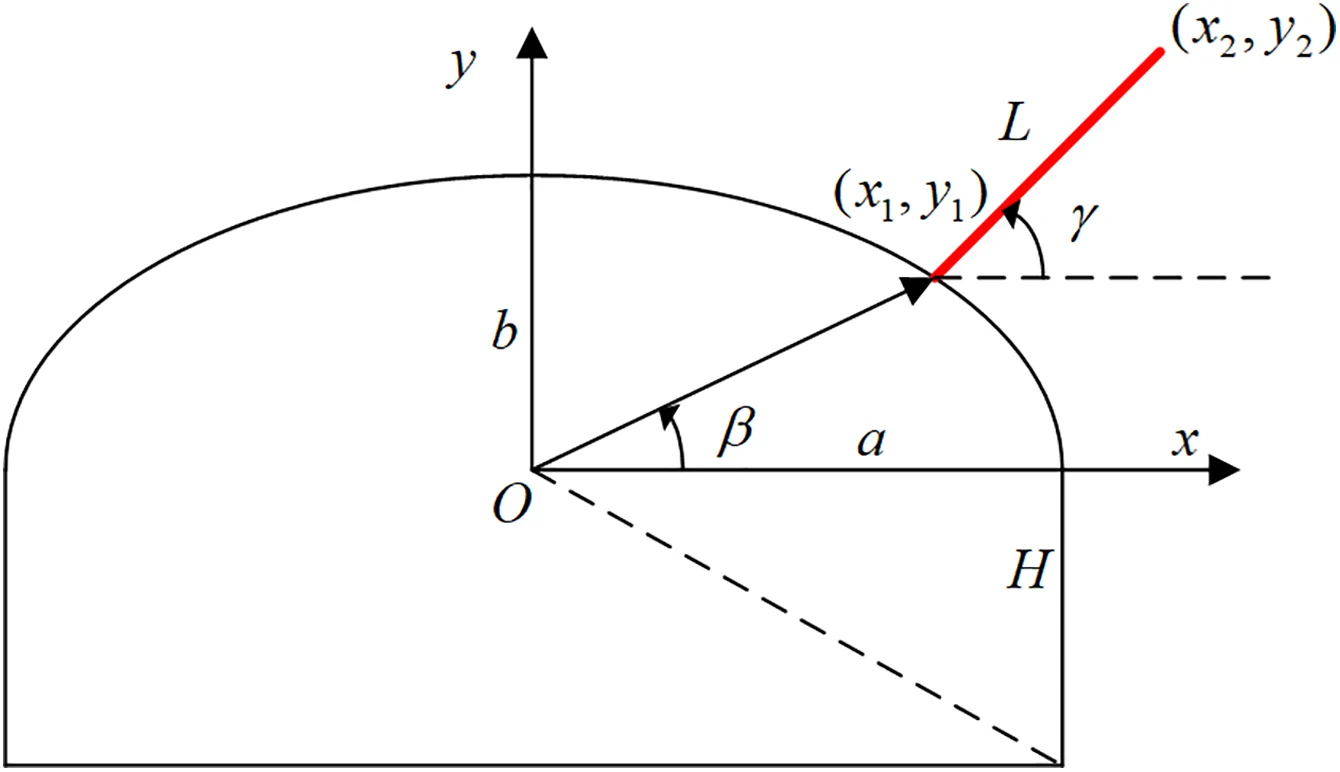
$0^{\circ }\leq \beta <180^{\circ }$.

**Fig 6:**
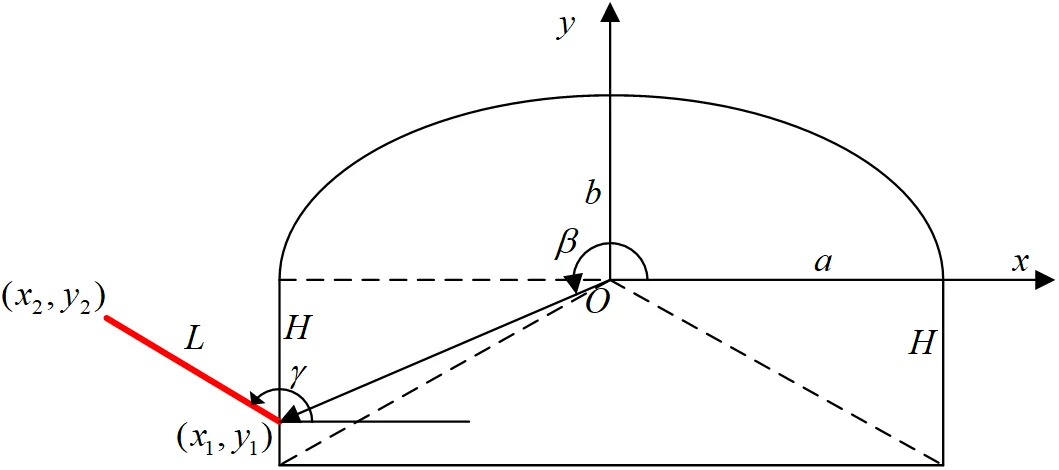
$(180^{\circ }\leq \beta <180^{\circ }+arctan\left(\frac{H}{a}\right)$.

As shown in Figs 4–6, the starting point of a cable is located at the edge of the roadway, and the length of a cable is L; then, the end point of a cable can be calculated on the basis of the cable length L, the starting point $\left(x_{\text{1}},y_{\text{1}}\right)$ of a cable and the inclination angle γ of a cable:


$$\begin{array}{c} \left\{ \begin{array}{c} @l@x_{\text{2}}=x_{\text{1}}+Lcos\gamma \\ y_{\text{2}}=y_{\text{1}}+Lsin\gamma \end{array}\right. \end{array}$$ (7)

Moreover, when the starting point $\left(x_{\text{1}},y_{\text{1}}\right)$ is fixed, the range of the inclination angle of the cable can be determined as follows:

When the starting point angle β is within the range of $-arctan\left(\frac{H}{a}\right)\leq \beta <{\text{0}}^{\circ }$ (Fig 4), the inclination angle of the cable is within the range of $-90^{\circ }\leq \gamma \leq 90^{\circ }$.

When the starting point angle β is within the range of ${\text{0}}^{\circ }\leq \beta <180^{\circ }$ (Fig 5), the range of the cable inclination angle is within the range of $-arctan\left(\frac{b^{\text{2}}x_{\text{1}}}{a^{\text{2}}y_{\text{1}}}\right)\leq \gamma \leq -arctan\left(\frac{b^{\text{2}}x_{\text{1}}}{a^{\text{2}}y_{\text{1}}}\right)+180^{\circ }$.

When the starting point angle β is within the range of $180^{\circ }\leq \beta <180^{\circ }+arctan\left(\frac{H}{a}\right)$ (Fig 6), the inclination angle of the cable is within the range of $90^{\circ }\leq \gamma \leq 270^{\circ }$.

The results of the analysis above reveal that the roadway water prevention and support variables have a length of ten $l_{\text{1}},l_{\text{2}},•••,l_{10}$, a cable number, a cable length L (to reduce the iteration time and for the convenience of engineering practice, all the cable lengths are equal), a starting point for the cable (all the starting points should be on the edge of the roadway), and an inclination angle for the cable. The objective function can be expressed as follows:


$$\begin{array}{c} f=A_{\text{grouting\_area}}\times L_{\text{all\_cable\_length}} \end{array}$$ (8)

Moreover, the roof and floor displacement should be less than or equal to 0.3 m, and the water flow into the roadway should be less than 0.2 m^3^/h. During the numerical simulation, if the roof and floor displacements are greater than 0.3 m or if the water flow into the roadway is greater than 0.2 m^3^/h, then the objective function value is set to infinity.

Therefore, the constraint condition and the objective function are determined, and the procedure for determining the optimal grouting area and cable distribution is as follows:

Step 1: Initialize the parameters of the firefly algorithm (FFA), in this paper, the population size is 50, randomization parameter α is 0.02, initial attractiveness at distance 0 $\beta_{\text{0}}$ is 1.0, the light absorption coefficient γ is 2.0, the maximum number of iterations max_iter is 1000, and these hyperparameters were selected based on the reference [24]. And the roadway support parameters (Table 1).

**Table 1:** The initialized variables of supported parameters.

Variables	Ranges
Grouting area (lengths of the ten lines are $l_{\text{1}},l_{\text{2}},•••,l_{10}$)	[2.7m, 10m]
Cable number	[3,20]
Cable start points $\left(x_{\text{1}},y_{\text{1}}\right)$	The cable start points $\left(x_{\text{1}},y_{\text{1}}\right)$ should be on the edge of the roadway, it should be noted that the cable start points number would be changed when the cable number varied.
Cable angle γ	starting point angle β is within the range of ${\text{0}}^{\circ }\leq \beta <180^{\circ }$, cable inclination angle is within the range of $-arctan\left(\frac{b^{\text{2}}x_{\text{1}}}{a^{\text{2}}y_{\text{1}}}\right)\leq \gamma \leq -arctan\left(\frac{b^{\text{2}}x_{\text{1}}}{a^{\text{2}}y_{\text{1}}}\right)+180^{\circ }$; starting point angle β is within the range of $180^{\circ }\leq \beta <180^{\circ }+arctan\left(\frac{H}{a}\right)$, cable inclination angle is within the range of $90^{\circ }\leq \gamma \leq 270^{\circ }$. The number cable angle would be changed when the cable number varied.

Step 2: Generate initial solutions for numerical simulation on the basis of the predefined variable ranges and hyperparameters of the FFA.

Step 3: Generate the FLAC3D command stream using the grouting area parameters and cable parameters (number of cables, cable length, and cable position), and then execute the numerical simulation. On the basis of the simulation results (roof-floor relative displacement and roadway water inflow), and calculate the objective value via Equation 8 if the following constraints are met: roof−floor displacement ≤ 0.3 m, two-sided wall displacement ≤0.3 m, and roadway water inflow ≤ 0.2 m^3^/h. Otherwise, the objective value shall be assigned the value calculated by Equation 8 plus 1e7.

Step 4: Adjust the roadway support variables using the FFA update mechanism.

Step 5: Check the termination condition: if it is satisfied, terminate the numerical simulation and proceed to Step 6; if not, return to Step 2 for iterative optimization.

Step 6: Terminate the algorithm, and output the optimal results.

To implement the above process, Python scripting was adopted to fully automate and control the numerical simulation workflow using the FFA framework. The simulation iterates until the termination condition is met, after which the optimal grouting area and cable design parameters are determined. A numerical simulation case is presented to verify the effectiveness of the proposed method.

### 2.4. Example for verifying the proposed algorithm

To validate the proposed method, a numerical simulation was conducted, and the roadway of the Banji Coal Mine in Anhui, China, was selected as an example. The buried depth of roadway is 636 m, the height of the roadway was 3.2 m, the width of the roadway was 5.4 m, the speed of water flow into the roadway was 97 m^3^/h, the relative displacement between the roof and floor was 1.36 m, and the relative displacement of the two sides was 0.83 m. The underground water locations are shown in Fig 7. Water flows into roadways, and the large displacement of the roadways seriously threatens daily production.

**Fig 7:**
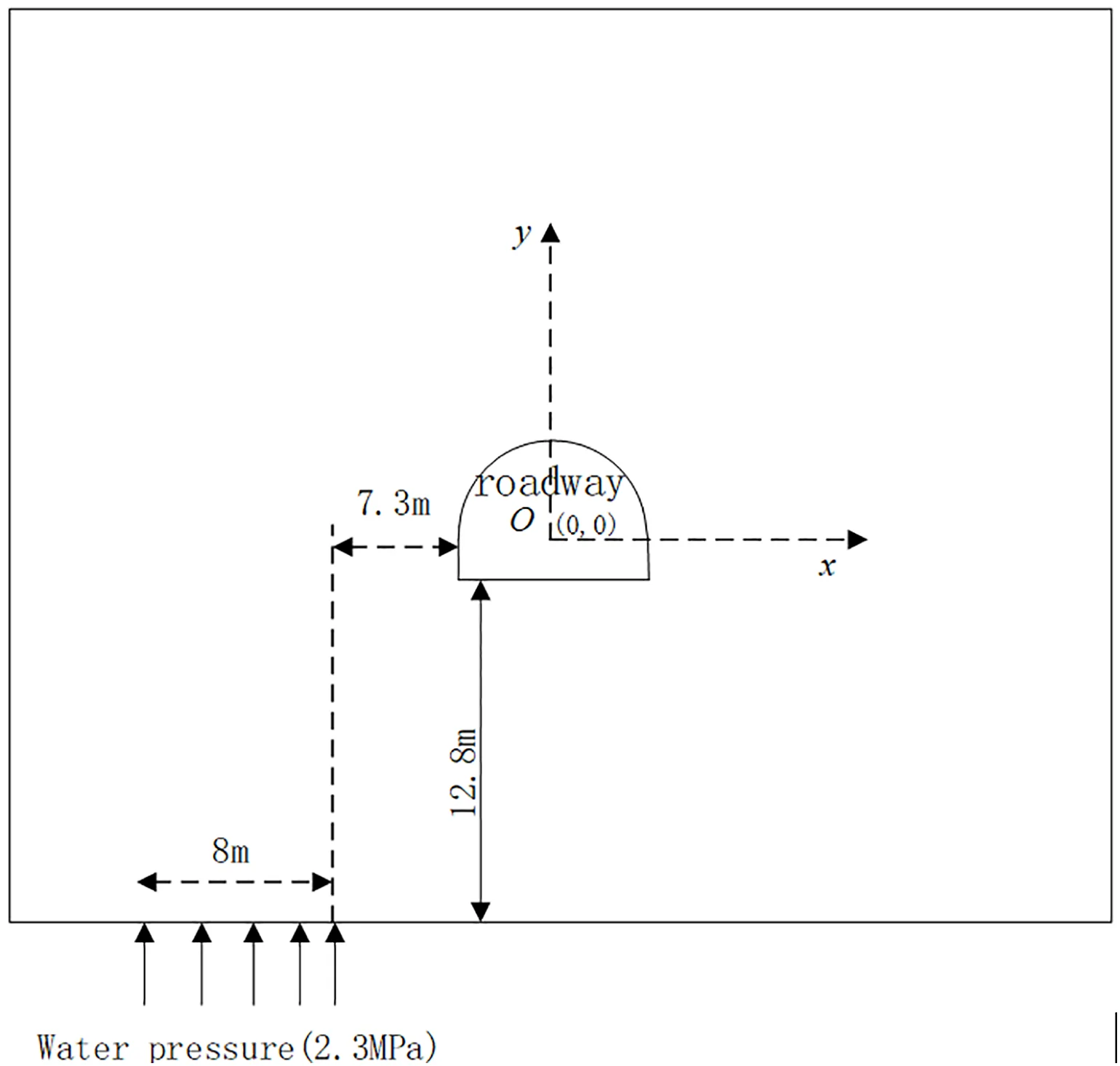
Underground water location and geometry of the numerical simulation model.

To determine the optimal cable location and grouting area, a FLAC3D numerical simulation model was constructed, the dimension of the numerical is 43.2m × 1m × 34.4m, and 35550 grid points and 17500 zones included in the numerical simulation model. And the centre coordinate of the roadway was the location of point (0, 0); the mechanical parameters of the rock mass and grouting area are listed in Table 2, these mechanical parameters were obtained from laboratory tests. In the numerical simulation, the Mohr-Colomb model was used.

**Table 2:** Mechanical parameters of the rock mass and grouting area.

	Density(kg/m^3^)	Bulk modulus(GPa)	Shear modulus (GPa)	Cohesion(MPa)	Friction angle (°)	Tension(MPa)
Rock mass	2573	9.5	3.1	1.2	9.2	0.6
Grouting area	2690	10.7	3.7	5.3	31.3	2.3

The permeability coefficient of the rock mass is 7.9e-11 m/s, whereas the permeability coefficient of the grouting area is 4.5e-13 m/s, and the porosity of rock mass is 0.45 and the grouting area is 0.43, and the Biot coefficient for rock mass is 0.85, and the grouting area is 0.75, and the fluid bulk is 1.3GPa, these mechanical parameters were obtained from laboratory tests, and the roadway surrounding rock surface is set as zero pore water pressure drainage boundary. A constant water pressure of 2.3 MPa is applied to the bottom aquifer within 8 m height to simulate deep confined water recharge. The top, left and right boundaries of the numerical model are all defined as impermeable no-flow boundaries. Groundwater flows steadily from the high-pressure bottom water-rich zone to the roadway drainage surface, and the stable water inflow is finally calculated.

Furthermore, the mechanical parameters of the cables are listed in Table 3, these mechanical parameters were obtained from laboratory tests.

**Table 3:** Mechanical parameters of the cables.

Young’s modulus (Pa)	201e9	Yield stress (KN)	562	Grout friction angle (°)	35
Poisson’s ratio	0.2	Grout stiffness (Pa)	1.32e7	Pretension(KN)	100
Cross-sectional area (m^2^)	396e-6	Grout cohesion (Pa)	1.95e5	Exposed perimeter(m)	0.0705

To accurately determine the most reasonable reinforcement scheme for the target structure, the firefly algorithm (FFA) was adopted for systematic optimization calculations. Through multiple iterations and simulation analysis based on the FFA algorithm, the optimal grouting area and the corresponding reasonable cable distribution layout were finally obtained. These key optimization results, including the specific range for parameters such as the grouting area and the detailed geometric parameters of the cables (such as diameter, spacing, and arrangement angle), are clearly listed in Table 4, Table 5, Figs 8–12 for reference and verification. Specifically, the optimization results indicate that the total number of cables required for the reinforcement scheme is 11, and each cable has a uniform length of 7.28 m, which is determined on the basis of the structural depth and demand for reinforcement to ensure sufficient bonding force and reinforcement effect. For more comprehensive and detailed parameter information, such as the specific coordinates of the grouting area, the cross-sectional parameters of the cables, and the relevant technical specifications, readers can refer to Table 4 and Table 5, where all the detailed parameters are systematically sorted and presented.

**Table 4:** Grouting length.

Series number	Value/m	Series number	Value/m
$l_{\text{1}}$	5.32	$l_{\text{6}}$	7.32
$l_{\text{2}}$	4.90	$l_{\text{7}}$	7.98
$l_{\text{3}}$	5.54	$l_{\text{8}}$	6.68
$l_{\text{4}}$	5.67	$l_{\text{9}}$	5.75
$l_{\text{5}}$	6.16	$l_{10}$	5.54

**Table 5:** Cable geometry parameters.

Series number	Cable length/m	Start point/m	Incline angle/°
1	7.28	(2.7, −0.99)	−10
2	7.28	(−2.7, −0.99)	191
3	7.28	(2.7, −0.33)	−5
4	7.28	(−2.7, −0.34)	185
5	7.28	(2.65, 0.39)	13
6	7.28	(−2.65, 0.40)	166
7	7.28	(1.84, 1.68)	32
8	7.28	(−1.90, 1.62)	147
9	7.28	(1.22, 2.05)	63
10	7.28	(−1.22, 2.06)	117
11	7.28	(0.01, 2.29)	91

**Fig 8:**
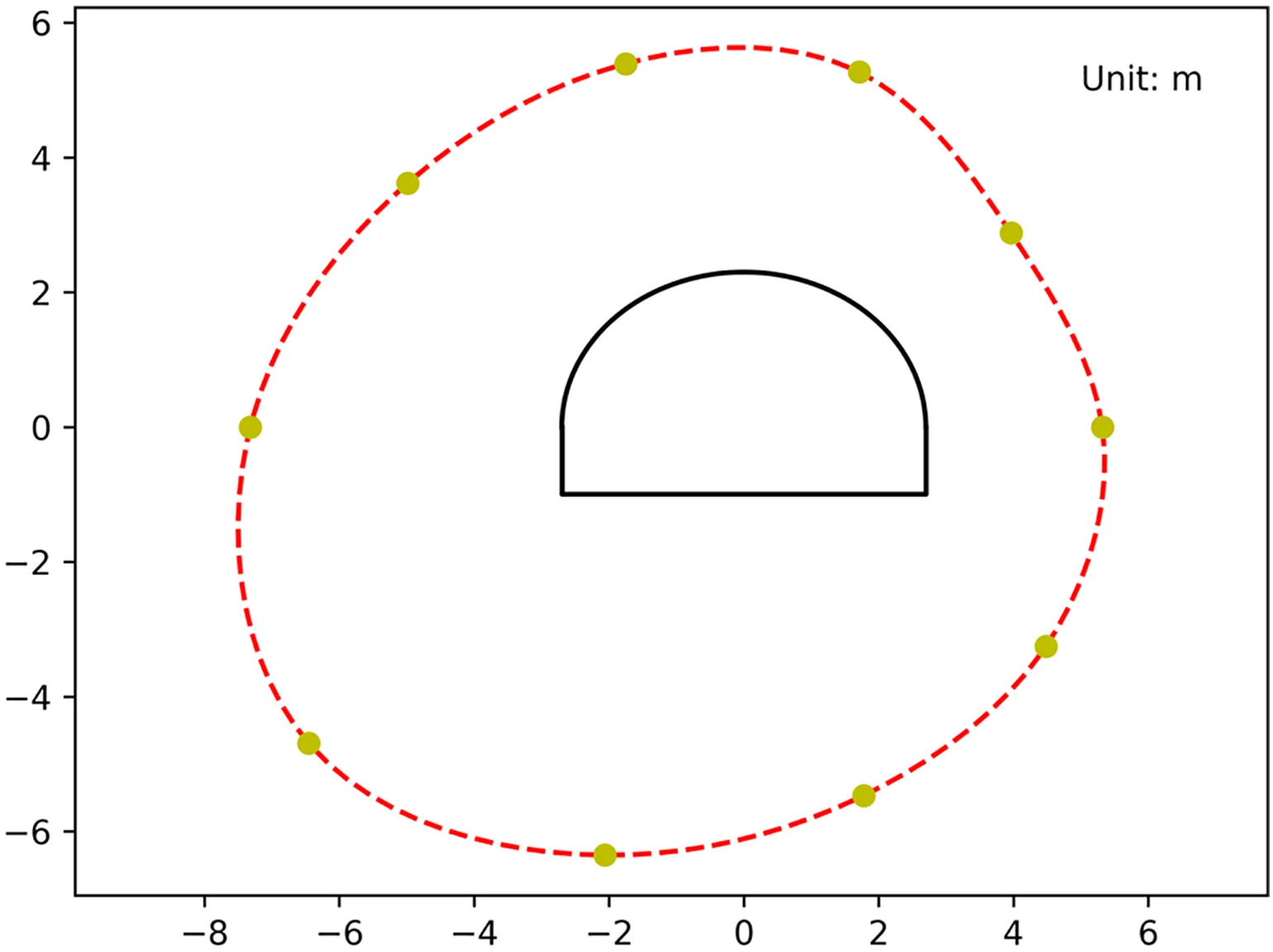
Outline of the grouting area.

**Fig 9:**
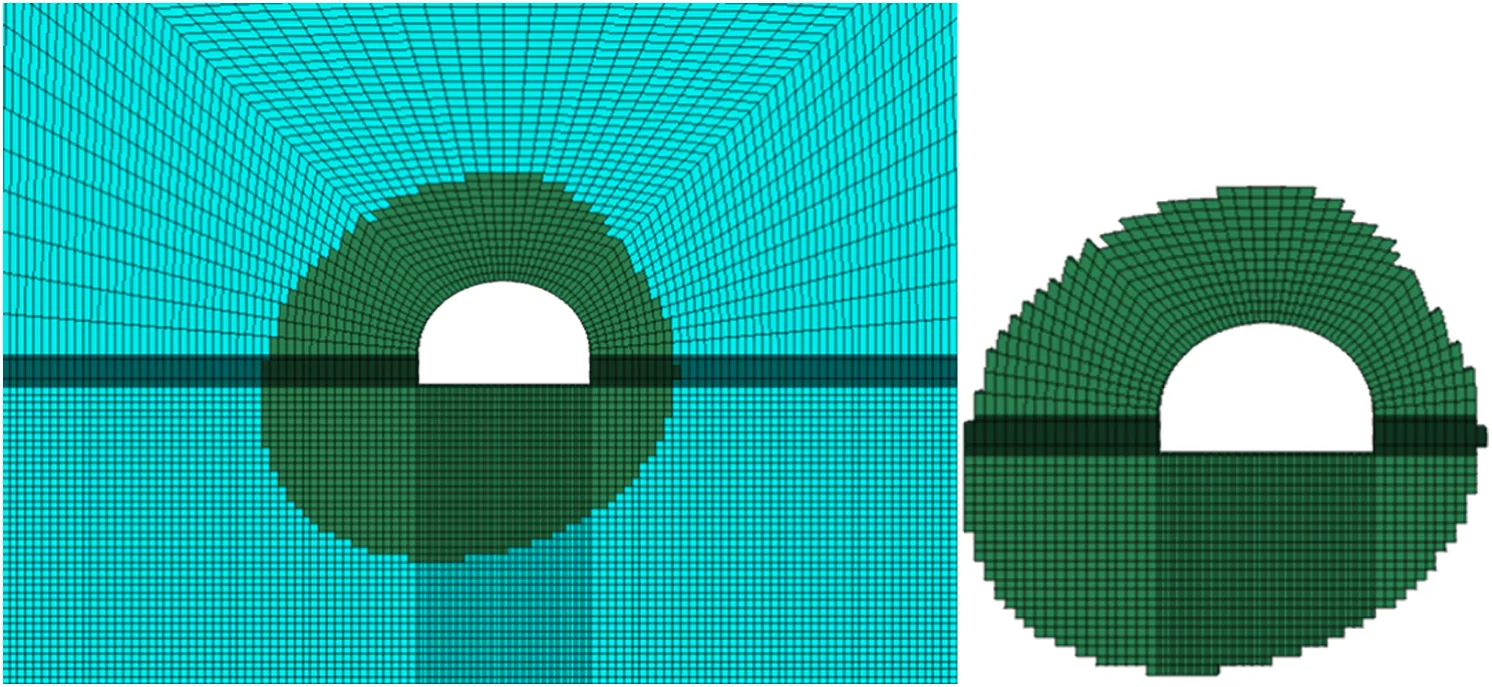
Grouting area in the numerical simulation model.

**Fig 10:**
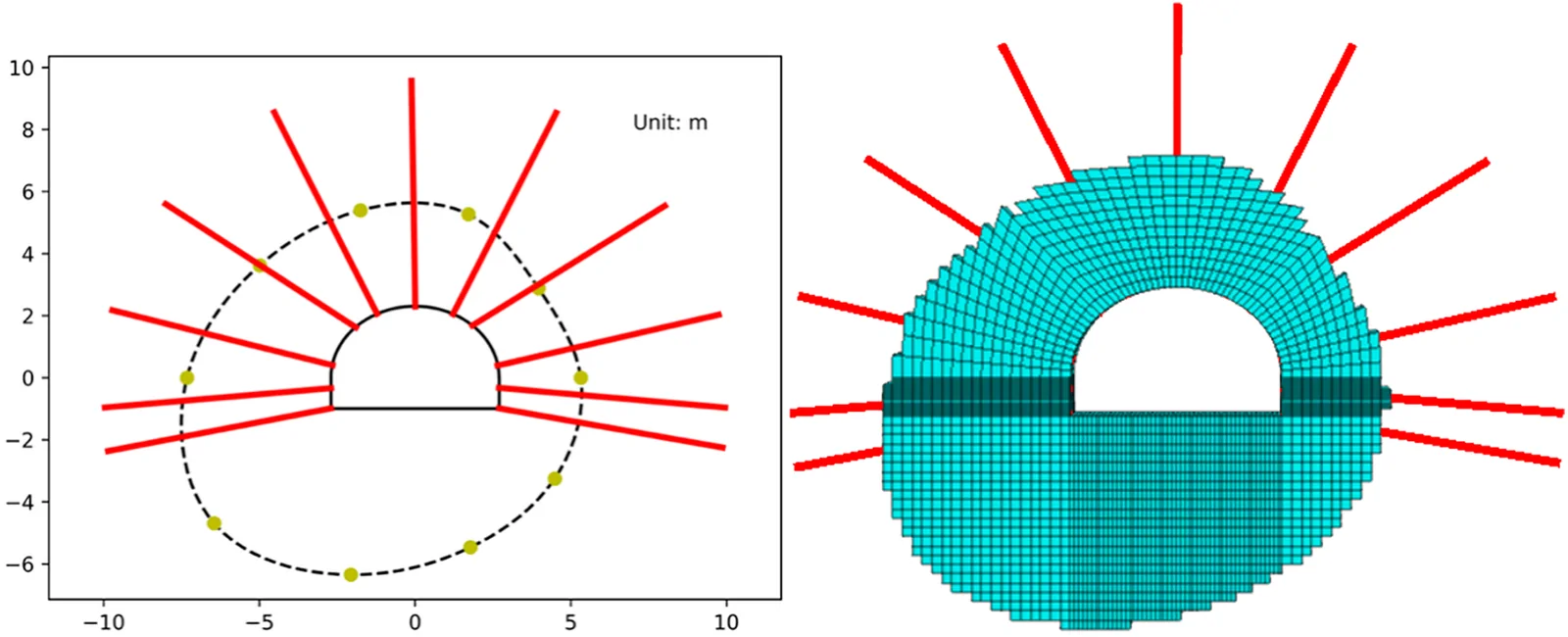
Cable distribution.

**Fig 11:**
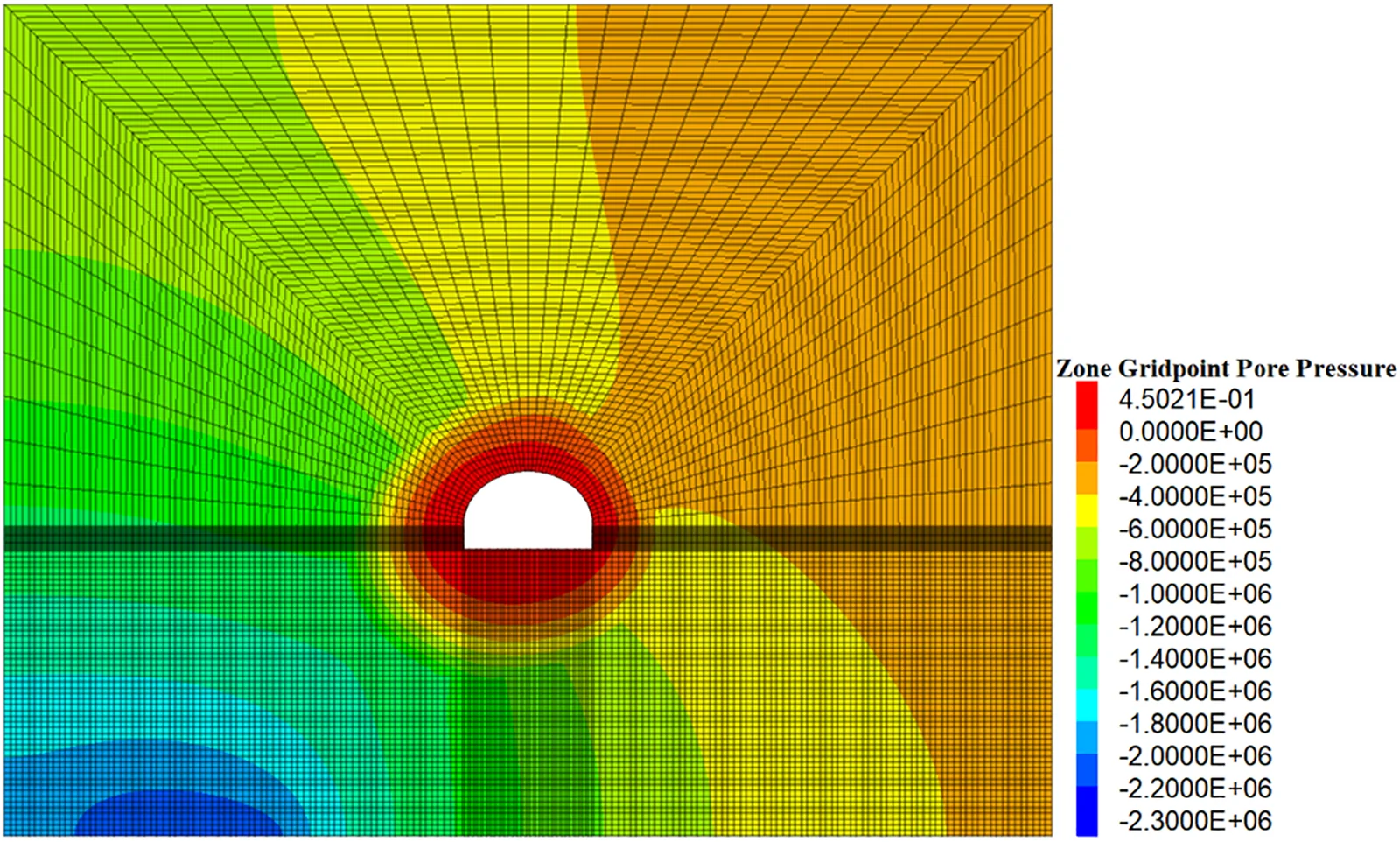
Pore pressure distribution after grouting.

**Fig 12:**
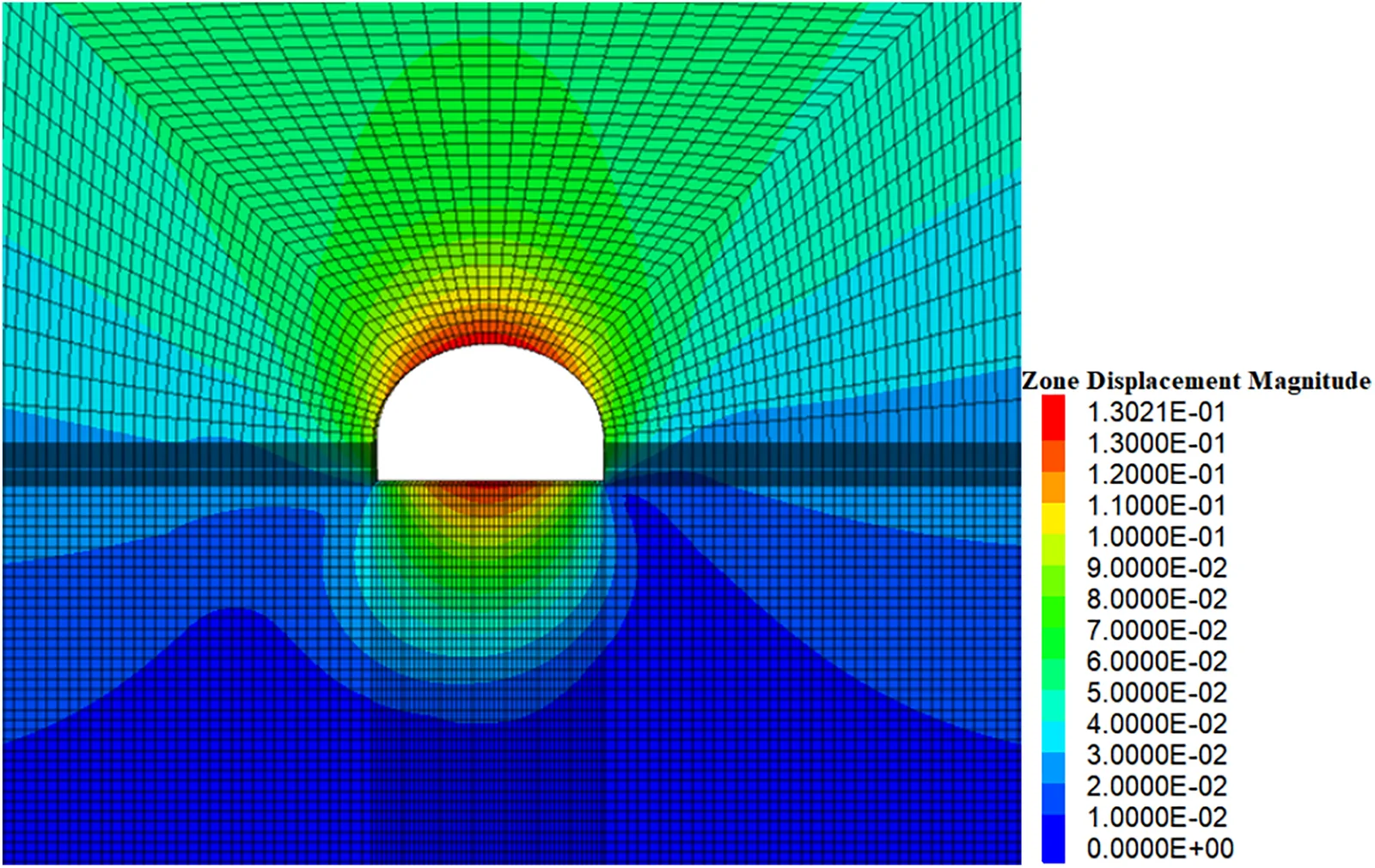
Deformation of the roadway with a support.

The numerical simulation results are systematically presented in Fig 11 and Fig 12. According to the simulation data, four points were determined to record the displacement of roadway(Fig 13), the relative vertical displacement between the roof and floor of the roadway is 0.27 m, whereas the relative horizontal displacement of the two sidewalls reaches 0.23 m. Both values are less than the allowable displacement specified in the relevant engineering standards, demonstrating satisfactory deformation control. In addition, the simulated rate of water inflow into the roadway is 0.169 m^3^/h, which is also below the critical threshold of 0.2 m^3^/h. The minimum objection function value is 8315.50m^2^▪m. Meanwhile, the total computation time is 26.4h, the computation time is acceptable. Furthermore, to verify the steadiness of the proposed method, the process was repeated for 10 times, and the convergence history was illustrated in Fig 14.

**Fig 13:**
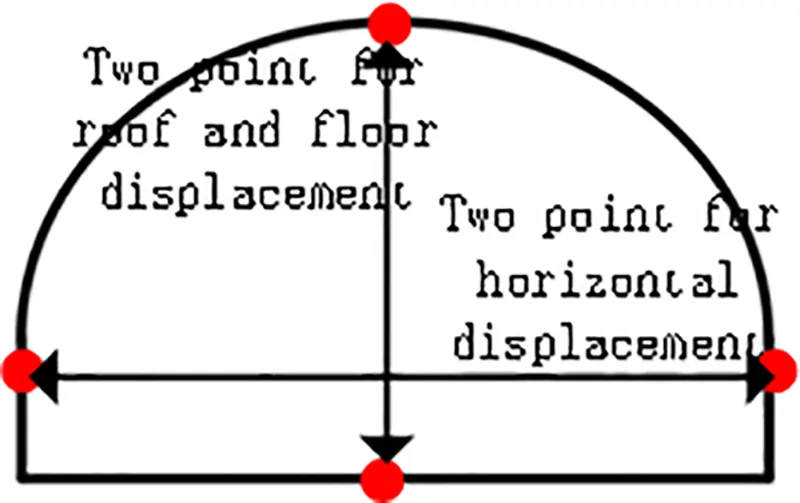
Deformation monitoring point.

**Fig 14:**
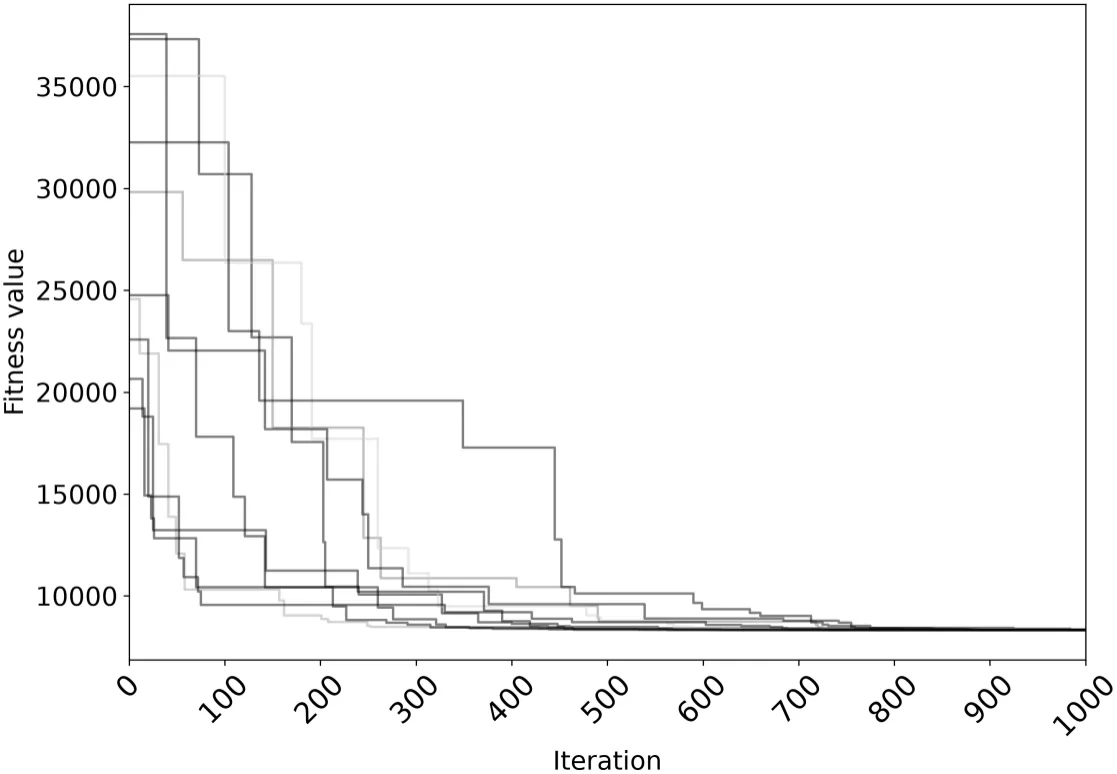
The convergence history of 10 repeated numerical simulation.

As illustrated in Fig 14, the final objective function value are quite close, the mean objective function value in Fig 14 is 8316.53 and the standard deviation value is 0.71, which means that the 10 objective value is quite close, and the minimum value of 10 results is 8315.50.

To further verify the accuracy and reliability of the numerical simulation outcomes, the grouting reinforcement scheme and cable arrangement design adopted in the simulation have been implemented in field engineering practice. The field monitoring data reveal that the actual water inflow into the roadway is 0.13 m^3^/h, the measured relative displacement between the roof and floor is 0.25 m, and the relative displacement of the two sides is 0.20 m, the displacement and the water flow inflow roadway were recorded for 3 months, hence, the recorded data was reliable. These field test results are highly consistent with the numerical simulation predictions. Both the numerical simulation analysis and onsite engineering verification demonstrate that the support and treatment method proposed in this study is rational and reliable. Therefore, this method can be effectively popularized and applied in similar underground engineering practices.

## 3. Discussion

Groundwater poses a significant threat to engineering construction, particularly in underground roadway mining. It not only disrupts normal construction progress but also leads to intractable safety hazards and economic losses. On the one hand, high-velocity groundwater inflow into roadways can directly trigger water inrush disasters, submerging equipment, blocking construction passages, endangering personnel safety, and even causing heavy casualties in severe cases. On the other hand, long-term groundwater seepage into rock pores and fractures severely reduces the strength, stiffness and integrity of the surrounding rock, weakens its bearing capacity, and further induces large deformation, floor heave, roof collapse or rib spalling. These issues delay construction and increase project maintenance costs. Therefore, scientific and effective measures are urgently required to control groundwater hazards in underground engineering.

Given the severe harm caused by groundwater in roadway engineering, numerous scholars worldwide have conducted extensive experimental research, theoretical analysis and engineering practice on groundwater control and roadway support schemes. Valuable achievements have been accumulated through long-term investigations, and various technical measures—including grouting reinforcement, drainage, waterproof curtains and diverse support structures—have been developed and applied to resist groundwater intrusion and control roadway deformation. Nevertheless, obvious shortcomings remain in current applications. For instance, over-support can ensure roadway stability and prevent deformation or collapse due to insufficient strength, yet it consumes excessive materials, labor and funds, resulting in poor economic efficiency. Conversely, inadequate support strength that fails to match the actual roadway stress and surrounding rock mechanical properties easily leads to excessive deformation and even collapse, bringing serious safety risks and economic losses. In most existing literature, researchers typically propose several alternative support schemes and select the so-called optimal one based on experience or simple comparison. Such a selection process lacks systematic theoretical guidance and scientific quantitative analysis, leading to non-scientific and subjective results that can hardly meet the demands of complex underground roadway engineering.

Accordingly, to resolve the problems of unreasonable support design and inefficient water control in underground roadway engineering, and to achieve optimal support and water prevention parameters for safe and economical construction, this study combines the firefly algorithm (FFA) with FLAC3D numerical simulation to establish a comprehensive optimization model for roadway support and seepage control. First, key factors influencing roadway stability and groundwater seepage were identified via theoretical analysis, and optimization objectives (including minimizing support cost, maximizing roadway stability and reducing seepage) as well as constraint conditions (such as support strength limits, waterproofing requirements and construction feasibility) were defined. Then, the FFA was adopted to optimize the initial support and water control parameters, while FLAC3D was used to simulate stress distribution, deformation characteristics and seepage behavior under different parameter combinations to verify their rationality. Through iterative algorithm optimization and numerical simulation, the optimal parameter combination for roadway support and seepage prevention was ultimately determined.

To further validate the effectiveness and practicality of the proposed method, an engineering case was adopted for verification. The optimized parameters and waterproof measures, after numerical verification, were applied in field practice. Engineering monitoring data show that numerical results are highly consistent with actual site conditions, demonstrating that the method is reliable, feasible and of good engineering application value. This research provides scientific theoretical guidance and practical references for the design of support and seepage prevention systems in underground roadways.

However, this study has several limitations to be addressed and supplemented in future research. Only cable support and grouting reinforcement were considered here, whereas actual underground engineering involves various supports (e.g., shotcrete, steel arch, and combined support) with distinct features and applicable scenarios. Future work will explore more support types, optimize the combination of multiple supports and anti-seepage measures, and account for complex geological conditions (variable rock properties, high ground stress, complex groundwater distribution). This will enhance the comprehensiveness, rigor, and applicability of the optimization method, supporting safer, more efficient, and economical underground engineering construction.

## 4. Conclusions

To accurately determine the optimal grouting area and reasonable cable distribution scheme in geotechnical engineering support design, in this study, an optimal algorithm was integrated with numerical simulation technology, and a systematic technical method for determining optimal support parameters was established. The main conclusions of this paper are summarized as follows:

(1) The firefly algorithm (FFA) and FLAC3D numerical simulation software were combined to form a complete technical framework for determining the optimal support parameters of the grouting and cables. By comparing and analysing the simulation results, the optimal combination of support parameters for ensuring the stability of the rock mass and minimizing the engineering cost was ultimately identified.
(2) To verify the validity, reliability, and engineering applicability of the proposed method (combining the FFA algorithm and FLAC3D numerical simulation), a specific engineering example was selected for verification. The proposed method was applied to an example project for calculation and optimization, and the optimal grouting area parameters and cable distribution scheme suitable for the example project were obtained. The obtained optimal support parameters were applied to an actual engineering example, and the results of comparison reveal that the onsite monitoring data are in good agreement with the numerical simulation results, with small deviations and high consistency. These findings indicate that the method proposed for determining optimal support parameters is scientific, reliable, and feasible and can provide effective technical support and reference for the design and construction of similar geotechnical engineering support projects.

## Supporting information

S1 Data Minimal dataset 1. (DOC)

S2 Data Minimal dataset 2. (TXT)
