# Supplementary material for: Optimal parameters of roadway supports for water prevention based on the FFA algorithm
Source: PLoS One. 2026 Oct 1;21(10):e0357258. doi: 10.1371/journal.pone.0357258 (PMC13630240; doi:10.1371/journal.pone.0357258)
Supplement: Supplementary file 1 [file pone.0357258.s001.doc]

Table 2 Mechanical parameters of the rock mass and grouting area

|  | Density(kg/m^3^) | Bulk modulus(GPa) | Shear modulus (GPa) | Cohesion(MPa) | Friction angle (°) | Tension(MPa) |
| --- | --- | --- | --- | --- | --- | --- |
| Rock mass | 2573 | 9.5 | 3.1 | 1.2 | 9.2 | 0.6 |
| Grouting area | 2690 | 10.7 | 3.7 | 5.3 | 31.3 | 2.3 |

Table 3 Mechanical parameters of the cables

| Young’s modulus (Pa) | 201e9 | Yield stress (KN) | 562 | Grout friction angle (°) | 35 |
| --- | --- | --- | --- | --- | --- |
| Poisson’s ratio | 0.2 | Grout stiffness (Pa) | 1.32e7 | Pretension(KN) | 100 |
| Cross-sectional area (m^2^) | 396e-6 | Grout cohesion (Pa) | 1.95e5 | Exposed perimeter(m) | 0.0705 |

Table 4 Grouting length

| Series number | Value/m | Series number | Value/m |
| --- | --- | --- | --- |
| $l_{1}$ | 5.32 | $l_{6}$ | 7.32 |
| $l_{2}$ | 4.90 | $l_{7}$ | 7.98 |
| $l_{3}$ | 5.54 | $l_{8}$ | 6.68 |
| $l_{4}$ | 5.67 | $l_{9}$ | 5.75 |
| $l_{5}$ | 6.16 | $l_{10}$ | 5.54 |

Table 5 Cable geometry parameters

| Series number | Cable length/m | Start point/m | Incline angle/° |
| --- | --- | --- | --- |
| 1 | 7.28 | (2.7, -0.99) | -10 |
| 2 | 7.28 | (-2.7, -0.99) | 191 |
| 3 | 7.28 | (2.7, -0.33) | -5 |
| 4 | 7.28 | (-2.7, -0.34) | 185 |
| 5 | 7.28 | (2.65, 0.39) | 13 |
| 6 | 7.28 | (-2.65, 0.40) | 166 |
| 7 | 7.28 | (1.84, 1.68) | 32 |
| 8 | 7.28 | (-1.90, 1.62) | 147 |
| 9 | 7.28 | (1.22, 2.05) | 63 |
| 10 | 7.28 | (-1.22, 2.06) | 117 |
| 11 | 7.28 | (0.01, 2.29) | 91 |
