# Supplementary material for: Optimal parameters of roadway supports for water prevention based on the FFA algorithm
Source: PLoS One. 2026 Oct 1;21(10):e0357258. doi: 10.1371/journal.pone.0357258 (PMC13630240; doi:10.1371/journal.pone.0357258)
Supplement: Supplementary file 2 [file pone.0357258.s004.doc]

Response

Reviewer #1: This study integrates firefly algorithm (FFA) with FLAC3D numerical simulation. Taking Banji Coal Mine roadway as the research object, FFA is adopted to optimize grouting range and anchor cable layout. Field application verifies that simulation results match practical data well. The study is meaningful and interesting, however, some questions and revision should be taken before the acceptance.
Response: Thanks for your comments, based on your comments, the page number and line number were added in the manuscript, meanwhile, the exact changes made in the revise manuscript, including corresponding page and line numbers were given.

(1)In this study, only one Coal Mine was taken as an example, the proposed method can be suitable for other coal mine
Response: Thanks for your comments, the proposed method can be applied to other coal mine, in the manuscript, once the shape of roadway and rock mechanic parameters were determined, the optimal grouting area and cable distribution can be determined based on the proposed method.

(2)The FFA algorithm was used to find the optimal parameters for water prevention, why this algorithm was selected? please explain the reasons.
Response: Thanks for your comments, many other optimization algorithm can be also used in the manuscript. In our manuscript, the FFA algorithm was selected, the FFA has several advantages: first, it features a simple and intuitive mechanism with few adjustable parameters, making it easy to understand, implement and program. Second, it exhibits strong global search capability, especially for multimodal optimization problems, as its attraction mechanism allows fireflies to automatically cluster into subgroups and effectively avoid premature convergence to local optima. Third, it achieves a good balance between global exploration and local exploitation by adjusting randomness and absorption coefficients. The detailed process of FFA algorithm is described below. The merits of the FFA algorithm was given in part 2.1 of the manuscript.

(3)In this paper, the FFA algorithm and FLAC3D numerical simulation combined by Python language, are you sure the Python can be used for FLAC3D numerical simulation?
Response: Thanks for your comments, the FLAC3D has two secondary development language, one is the FISH language and the other one is the Python script language, the Python language was supported since Version 5.0, which can be seen the manual of FLAC3D.

(4)The obtained numerical simulation results were applied to engineering practice, the difference between numerical simulation and engineering practice, how do you determine the mechanical parameters of rock mass in numerical simulation.
Response: Thanks for your comments, in our manuscript, the rock mass mechanical parameters were obtained from physical tests, and then the rock mass mechanical parameters were calibrated based on the rock mass grade. It should be noted that numerical simulation results agree well with engineering practice, which indicated that the rock mass mechanical parameters applied to numerical simulation model is reliable.

(5) How do you determine the range of cable length in the manuscript.
    Response: Thanks for your comments, in our manuscript, the range of cable length is within the range of 0.1m and 10m, in fact, wider of the range cable length can be more capable to obtain the optimal cable length, to reduce the iteration time, hence, the range (0.1, 10) was selected.

This paper can be accepted after minor revision.

Response: Thank you again. There is no questions needed to be answered.

Reviewer #2: The manuscript titled “Optimal Parameters of Roadway Supports for Water Prevention based on the FFA Algorithm” combines the firefly algorithm (FFA) with FLAC3D fluid–mechanical simulation to determine the optimal grouting area and cable layout of a water-bearing roadway, and the optimized scheme was applied to the Banji Coal Mine. The topic is of practical value in mining engineering, and the field implementation is a strength that many optimization studies do not have. However, several material parameters and boundary conditions are physically inadmissible or contradictory, and most of the information needed to reproduce the work is missing, so the results cannot be assessed in the current version. Please see my comments below:
Response: Thanks for your comments, based on your comments, the page number and line number were added in the manuscript, meanwhile, the exact changes made in the revise manuscript, including corresponding page and line numbers were given.

1. In Table 1, the bulk modulus (3.1 GPa) is much smaller than the shear modulus (9.5 GPa), which gives a negative Poisson’s ratio for the rock mass (ν = −0.26). The grouting area gives ν = −0.24. This is not possible for coal measure rock. If the two columns are exchanged, ν ≈ 0.35, which is reasonable. Please check the laboratory data, correct Table 1 and re-run all the simulations, because every mechanical result depends on these values.
Response: Thanks for your comments, it is our mistakes, the two columns should be exchanged, our numerical simulation are correct, there exist some mistakes when writing the manuscript, there is no need to re-run all the simulations. The changes were highlighted by red color, which can be seen in the revised manuscript.

2. The seepage boundary condition is contradictory. The text says the model boundary is a waterproof boundary, the water pressure at the roadway edge is 0 MPa, and the flow is steady, but Fig. 3 shows a water pressure of 2.3 MPa applied at the bottom over 8 m. If all outer boundaries are impermeable and the roadway is a drain, the steady-state inflow should be zero, and the reported 0.169 m³/h cannot be obtained. Please give the complete hydraulic boundary conditions and make the text consistent with Fig. 3.
Response: Thanks for your comments, based on your comments, the model boundary of the numerical simulation was changed. In our manuscript, the roadway surrounding rock surface is set as zero pore water pressure drainage boundary. A constant water pressure of 2.3 MPa is applied to the bottom aquifer within 8 m height to simulate deep confined water recharge. The top, left and right boundaries of the numerical model are all defined as impermeable no-flow boundaries. Groundwater flows steadily from the high-pressure bottom water-rich zone to the roadway drainage surface, and the stable water inflow is finally calculated. The changes were highlighted by red color, which can be seen in the revised manuscript.

3. The dimension of the optimization problem is not consistent. Section 2.3 lists ten grouting lengths, the cable number, the cable length, and the starting point and inclination angle of each cable, which is at least 34 variables for 11 cables, but Step 1 of the same section says the variable is ten-dimensional. Please give the variable vector, its dimension, and the search range of each variable in a table.
Response: Thanks for your comments, based on your comments, the initialized parameters and ranges were given in Table 1, the changes were highlighted by red color, and the changed can be seen in the revised manuscript.

4. The parameters of the FFA are not reported. The population size, α, β₀, γ and max_iter actually used are not given, only the general ranges in Section 2.1, and there is no convergence curve, iteration number, total number of FLAC3D calls, or computation time. As FFA is a stochastic algorithm, please add the hyperparameters, the convergence history, and several independent runs with the mean and scatter of the best objective value.
Response: Thanks for your comments, based on your comments, in this paper, for the FFA algorithm, the parameters were set as follows: Initialize the parameters of the firefly algorithm (FFA), in this paper, the population size is 50, randomization parameter $\alpha$is 0.02, initial attractiveness at distance 0 $\beta_{0}$ is 1.0, the light absorption coefficient $\gamma$ is 2.0, the maximum number of iterations $max\_iter$ is 1000, the changes were added in the revised manuscript. Meanwhile, based on your comments, the convergence history, and the mean and scatter of the best objective value was given in the manuscript.

5. The numerical model is not described. Please add the model dimensions, element size, number of zones, and a mesh sensitivity analysis. The porosity, Biot coefficient and fluid bulk modulus required by Equations (10)–(13) are not given, and the unit of the permeability coefficient (7.9e−11 and 4.5e−13) is missing, so these values cannot be interpreted.
Response: Thanks for your comments, based on your comments, the numerical simulation model is described, the model dimensions, element size, number of zones were given, meanwhile, the porosity, Biot coefficient and fluid bulk modulus are given, which can be seen in the revised manuscript.

6.In Section 2.3, Step 3, the objective value of an infeasible solution is set to infinity. If most of the initial population is infeasible, all fireflies have the same brightness, the attraction term disappears, and the algorithm degenerates into a random search. Please report the feasible proportion of the initial population, and consider a graded penalty function or a repair operator.
Response: Thanks for your comments, if the condition is not satisfied, the objective value shall be assigned the value calculated by Equation (16) plus 1e7.

7. Section 2.2 repeats the FLAC3D theory manual (Reference [21]) at too much length. Equations (6)–(13) are presented but the parameters they introduce (α, φ, K_f, M_b) are never assigned values or used afterwards. Please shorten this section and keep only the formulation and parameters actually adopted, with the source clearly cited.
Response: Thanks for your comments, based on your comments, this part was shorten, which can be seen in the revised manuscript.

8. The grouting area is described by ten rays at 36° interval, but the reason for choosing ten is not explained and no sensitivity analysis of the number of control points is given. The optimized boundary in Table 3 (4.90–7.98 m) is close to an ellipse, so it is not clear how much the ten parameters contribute. Please also discuss whether such a shape can actually be constructed by borehole grouting.
Response: Thanks for your comments, as a matter of fact, more accuracy of the numerical results would be obtained if more end points selected. If more end points were used, then more variables needed to be determined during the process, in our manuscript, ten end points were determined based on the experience, meanwhile, the end points were connected by cubic spline interpolation method, the closed line is smooth, and the results were acceptable. However, the actual grouting depth of roadways after grouting can be comprehensively determined by construction parameters, on-site verification and non-destructive testing. The slurry diffusion range is preliminarily judged according to the final drilling depth, changes of grouting pressure and flow rate, and slurry consumption. After the slurry solidifies, core sampling and borehole inspection are conducted through inspection holes arranged between grouting holes to directly detect the maximum effective filling depth of slurry veins. Meanwhile, geophysical methods such as ground penetrating radar and acoustic wave detection are adopted to define the grouting reinforcement boundary, and the actual effective grouting depth of roadways is finally confirmed by mutual verification of various data. I hope my answer can meet your satisfactory.

9. The water inflow decreases from 97 m³/h to 0.13 m³/h, about 750 times, while the permeability ratio of the rock mass and the grouting area is only about 176. Please give a simple consistency check of this reduction against the grouting geometry.
Response: Thanks for your comments, The discrepancy is mainly caused by the change of seepage flow channel geometry after grouting. Grouting not only reduces rock mass permeability, but also greatly narrows, blocks and disconnects original dominant seepage paths. The actual seepage cross-sectional area decreases sharply, leading to a far larger multiple of water inflow reduction than the permeability ratio, which is consistent with the field grouting geometric characteristics. Meanwhile, the numerical simulation was repeated, the numerical simulation results were right.

10. In Section 2.4, “The roadway height was less than 636 m, the height of the roadway was 3.2 m” is contradictory in one sentence. The first value should be the burial depth.
Response: Thanks for your comments, the contradictory part was revised, which can be seen in the revised manuscript.

11. In Table 2, “Yield tension (Pa) 1420e6” is a stress, not a tension. In FLAC3D the cable yield tension is a force; with the cross-sectional area of 396e−6 m² it should be about 562 kN. The pretension and the exposed perimeter of the cable are also not given.
Response: Thanks for your comments, the unit of yield tension is revised, the pretension and the exposed perimeter of the cable are added in the manuscript.

12. In Section 2.1, the FFA is said to be proposed by Yang in 2008, but Reference [20] is dated 2010. Reference [1] and several other entries are also not in the correct format, and References [12], [13], [18] and [19], which are about Schmidt hammer tests and DEM parameter calibration, are not related to roadway support. Please check the whole reference list.
Response: Thanks for your comments, FFA is said to be proposed by Yang in 2010, which can be seen in the revised manuscript, and references [12], [13], [18] and [19] were deleted, and whole reference list were checked.

13. In Section 2.3, “As shown in Fig., the starting point of a cable …” the figure number is missing. In Section 2.4, Figs. 5–8 are listed but Fig. 4 is omitted, and the caption of Fig. 1(b) is separated from the figure.
Response: Thanks for your comments, in section 2.3, the figure number is added. Meanwhile, the Fig.4 is added. The Fig. 1 in the manuscript was revised.

14. Some references should be added to enrich the research background. For example, Combined effects of fault geometry and roadway cross-section shape on the collapse behaviors of twin roadways: an experimental investigation, Failure mechanism and support technology of weakly cemented soft rock roadway in water-rich environment, Asymmetric large deformation in deep mining-damaged roadways: mechanism and collaborative control technology by means of destressing-bolting.
Response: Thanks for your comments, based on your comments, some references were added to enrich the research background.

15. The English of the manuscript should be polished by a professional editor. Several sentences are repeated word by word, and some expressions are not standard.

Response: Thanks for your comments, based on your comments, our manuscript was polished by American Journal Expert, the editing proof was attached. The changed part was highlighted by red color, which can be seen in the revised manuscript.
