# Supplementary material for: Optimal parameters of roadway supports for water prevention based on the FFA algorithm
Source: PLoS One. 2026 Oct 1;21(10):e0357258. doi: 10.1371/journal.pone.0357258 (PMC13630240; doi:10.1371/journal.pone.0357258)
Supplement: Peer review history

# Peer review: Decision Letter 0

**DOI:** 10.1371/journal.pone.0357258.r001

**Authors:** Zhenhua Li

23 Jul 2026

Dear Dr. xu,

Thank you for submitting your manuscript to PLOS ONE. After careful consideration, we feel that it has merit but does not fully meet PLOS ONE’s publication criteria as it currently stands. Therefore, we invite you to submit a revised version of the manuscript that addresses the points raised during the review process.

Please submit your revised manuscript by Sep 21 2026 11:59PM. If you will need more time than this to complete your revisions, please reply to this message or contact the journal office at plosone@plos.org. When you're ready to submit your revision, log on to https://www.editorialmanager.com/pone/ and select the 'Submissions Needing Revision' folder to locate your manuscript file.

- A letter that responds to each point raised by the academic editor and reviewer(s). You should upload this letter as a separate file labeled 'Response to Reviewers'.
- A marked-up copy of your manuscript that highlights changes made to the original version. You should upload this as a separate file labeled 'Revised Manuscript with Track Changes'.
- An unmarked version of your revised paper without tracked changes. You should upload this as a separate file labeled 'Manuscript'.

If you would like to make changes to your financial disclosure, please include your updated statement in your cover letter. Guidelines for resubmitting your figure files are available below the reviewer comments at the end of this letter.

If applicable, we recommend that you deposit your laboratory protocols in protocols.io to enhance the reproducibility of your results. Protocols.io assigns your protocol its own identifier (DOI) so that it can be cited independently in the future. For instructions see: https://journals.plos.org/plosone/s/submission-guidelines#loc-laboratory-protocols. Additionally, PLOS ONE offers an option for publishing peer-reviewed Lab Protocol articles, which describe protocols hosted on protocols.io. Read more information on sharing protocols at https://plos.org/protocols?utm_medium=editorial-email&utm_source=authorletters&utm_campaign=protocols.

As the corresponding author, your ORCID iD is verified in the submission system and will appear in the published article. PLOS supports the use of ORCID, and we encourage all coauthors to register for an ORCID iD and use it as well. Please encourage your coauthors to verify their ORCID iD within the submission system before final acceptance, as unverified ORCID iDs will not appear in the published article. *Only*  the individual author can complete the verification step; PLOS staff *cannot*  verify ORCID iDs on behalf of authors.

We look forward to receiving your revised manuscript.

Kind regards,

Zhenhua Li

Academic Editor

PLOS One

Journal Requirements:

When submitting your revision, we need you to address these additional requirements.

1. Please ensure that your manuscript meets PLOS ONE's style requirements, including those for file naming. The PLOS ONE style templates can be found at

https://journals.plos.org/plosone/s/file?id=wjVg/PLOSOne_formatting_sample_main_body.pdf and

https://journals.plos.org/plosone/s/file?id=ba62/PLOSOne_formatting_sample_title_authors_affiliations.pdf

2. Please note that PLOS One has specific guidelines on code sharing for submissions in which author-generated code underpins the findings in the manuscript. In these cases, we expect all author-generated code to be made available without restrictions upon publication of the work. Please review our guidelines at https://journals.plos.org/plosone/s/materials-and-software-sharing#loc-sharing-code and ensure that your code is shared in a way that follows best practice and facilitates reproducibility and reuse.

3. Please update your submission to use the PLOS LaTeX template. The template and more information on our requirements for LaTeX submissions can be found at http://journals.plos.org/plosone/s/latex.

4. Thank you for stating the following in your Competing Interests section:

“no”

Please complete your Competing Interests on the online submission form to state any Competing Interests. If you have no competing interests, please state "The authors have declared that no competing interests exist.", as detailed online in our guide for authors at http://journals.plos.org/plosone/s/submit-now

This information should be included in your cover letter; we will change the online submission form on your behalf.

5. Please provide a complete Data Availability Statement in the submission form, ensuring you include all necessary access information or a reason for why you are unable to make your data freely accessible. If your research concerns only data provided within your submission, please write "All data are in the manuscript and/or supporting information files" as your Data Availability Statement.

6. PLOS requires an ORCID iD for the corresponding author in Editorial Manager on papers submitted after December 6th, 2016. Please ensure that you have an ORCID iD and that it is validated in Editorial Manager. To do this, go to ‘Update my Information’ (in the upper left-hand corner of the main menu), and click on the Fetch/Validate link next to the ORCID field. This will take you to the ORCID site and allow you to create a new iD or authenticate a pre-existing iD in Editorial Manager.

7. Thank you for stating the following in the Competing Interests section:

“no”

We note that one or more of the authors are employed by a commercial company: “China Coal Underground Space Technology (Shenzhen) Co., Ltd.”

1. Please provide an amended Funding Statement declaring this commercial affiliation, as well as a statement regarding the Role of Funders in your study. If the funding organization did not play a role in the study design, data collection and analysis, decision to publish, or preparation of the manuscript and only provided financial support in the form of authors' salaries and/or research materials, please review your statements relating to the author contributions, and ensure you have specifically and accurately indicated the role(s) that these authors had in your study. You can update author roles in the Author Contributions section of the online submission form.

Please also include the following statement within your amended Funding Statement.

“The funder provided support in the form of salaries for authors [insert relevant initials], but did not have any additional role in the study design, data collection and analysis, decision to publish, or preparation of the manuscript. The specific roles of these authors are articulated in the ‘author contributions’ section.”

If your commercial affiliation did play a role in your study, please state and explain this role within your updated Funding Statement.

2. Please also provide an updated Competing Interests Statement declaring this commercial affiliation along with any other relevant declarations relating to employment, consultancy, patents, products in development, or marketed products, etc.

Within your Competing Interests Statement, please confirm that this commercial affiliation does not alter your adherence to all PLOS ONE policies on sharing data and materials by including the following statement: "This does not alter our adherence to  PLOS ONE policies on sharing data and materials.” (as detailed online in our guide for authors http://journals.plos.org/plosone/s/competing-interests) . If this adherence statement is not accurate and  there are restrictions on sharing of data and/or materials, please state these. Please note that we cannot proceed with consideration of your article until this information has been declared.

Please include both an updated Funding Statement and Competing Interests Statement in your cover letter. We will change the online submission form on your behalf.

8. Please upload a new copy of Figure 3 as the detail is not clear. Please follow the link for more information:  https://journals.plos.org/plosone/s/figures

9. Please ensure that you refer to Figure 4 in your text as, if accepted, production will need this reference to link the reader to the figure.

10. If the reviewer comments include a recommendation to cite specific previously published works, please review and evaluate these publications to determine whether they are relevant and should be cited. There is no requirement to cite these works unless the editor has indicated otherwise.

Additional Editor Comments:

You will see from the referees' comments that additional information needs to be provided, and we ask that this be provided, before we consider you manuscript further.

[Note: HTML markup is below. Please do not edit.]

Reviewers' comments:

Reviewer's Responses to Questions

**Comments to the Author**

1. Is the manuscript technically sound, and do the data support the conclusions?

Reviewer #1: Yes

Reviewer #2: Yes

**********

2. Has the statistical analysis been performed appropriately and rigorously?

Reviewer #1: Yes

Reviewer #2: Yes

**********

3. Have the authors made all data underlying the findings in their manuscript fully available?

The PLOS Data policy

Reviewer #1: Yes

Reviewer #2: Yes

**********

4. Is the manuscript presented in an intelligible fashion and written in standard English?

Reviewer #1: Yes

Reviewer #2: Yes

**********

Reviewer #1: This study integrates firefly algorithm (FFA) with FLAC3D numerical simulation. Taking Banji Coal Mine roadway as the research object, FFA is adopted to optimize grouting range and anchor cable layout. Field application verifies that simulation results match practical data well. The study is meaningful and interesting, however, some questions and revision should be taken before the acceptance.

(1)In this study, only one Coal Mine was taken as an example, the proposed method can be suitable for other coal mine

(2)The FFA algorithm was used to find the optimal parameters for water prevention, why this algorithm was selected? please explain the reasons.

(3)In this paper, the FFA algorithm and FLAC3D numerical simulation combined by Python language, are you sure the Python can be used for FLAC3D numerical simulation?

(4)The obtained numerical simulation results were applied to engineering practice, the difference between numerical simulation and engineering practice, how do you determine the mechanical parameters of rock mass in numerical simulation.

(5)How do you determine the range of cable length in the manuscript.

This paper can be accepted after minor revision.

Reviewer #2: The manuscript titled “Optimal Parameters of Roadway Supports for Water Prevention based on the FFA Algorithm” combines the firefly algorithm (FFA) with FLAC3D fluid–mechanical simulation to determine the optimal grouting area and cable layout of a water-bearing roadway, and the optimized scheme was applied to the Banji Coal Mine. The topic is of practical value in mining engineering, and the field implementation is a strength that many optimization studies do not have. However, several material parameters and boundary conditions are physically inadmissible or contradictory, and most of the information needed to reproduce the work is missing, so the results cannot be assessed in the current version. Please see my comments below:

1. In Table 1, the bulk modulus (3.1 GPa) is much smaller than the shear modulus (9.5 GPa), which gives a negative Poisson’s ratio for the rock mass (ν = −0.26). The grouting area gives ν = −0.24. This is not possible for coal measure rock. If the two columns are exchanged, ν ≈ 0.35, which is reasonable. Please check the laboratory data, correct Table 1 and re-run all the simulations, because every mechanical result depends on these values.

2. The seepage boundary condition is contradictory. The text says the model boundary is a waterproof boundary, the water pressure at the roadway edge is 0 MPa, and the flow is steady, but Fig. 3 shows a water pressure of 2.3 MPa applied at the bottom over 8 m. If all outer boundaries are impermeable and the roadway is a drain, the steady-state inflow should be zero, and the reported 0.169 m³/h cannot be obtained. Please give the complete hydraulic boundary conditions and make the text consistent with Fig. 3.

3. The dimension of the optimization problem is not consistent. Section 2.3 lists ten grouting lengths, the cable number, the cable length, and the starting point and inclination angle of each cable, which is at least 34 variables for 11 cables, but Step 1 of the same section says the variable is ten-dimensional. Please give the variable vector, its dimension, and the search range of each variable in a table.

4. The parameters of the FFA are not reported. The population size, α, β₀, γ and max_iter actually used are not given, only the general ranges in Section 2.1, and there is no convergence curve, iteration number, total number of FLAC3D calls, or computation time. As FFA is a stochastic algorithm, please add the hyperparameters, the convergence history, and several independent runs with the mean and scatter of the best objective value.

5. The numerical model is not described. Please add the model dimensions, element size, number of zones, and a mesh sensitivity analysis. The porosity, Biot coefficient and fluid bulk modulus required by Equations (10)–(13) are not given, and the unit of the permeability coefficient (7.9e−11 and 4.5e−13) is missing, so these values cannot be interpreted.

6. In Section 2.3, Step 3, the objective value of an infeasible solution is set to infinity. If most of the initial population is infeasible, all fireflies have the same brightness, the attraction term disappears, and the algorithm degenerates into a random search. Please report the feasible proportion of the initial population, and consider a graded penalty function or a repair operator.

7. Section 2.2 repeats the FLAC3D theory manual (Reference [21]) at too much length. Equations (6)–(13) are presented but the parameters they introduce (α, ϕ, K_f, M_b) are never assigned values or used afterwards. Please shorten this section and keep only the formulation and parameters actually adopted, with the source clearly cited.

8. The grouting area is described by ten rays at 36° interval, but the reason for choosing ten is not explained and no sensitivity analysis of the number of control points is given. The optimized boundary in Table 3 (4.90–7.98 m) is close to an ellipse, so it is not clear how much the ten parameters contribute. Please also discuss whether such a shape can actually be constructed by borehole grouting.

9. The water inflow decreases from 97 m³/h to 0.13 m³/h, about 750 times, while the permeability ratio of the rock mass and the grouting area is only about 176. Please give a simple consistency check of this reduction against the grouting geometry.

10. In Section 2.4, “The roadway height was less than 636 m, the height of the roadway was 3.2 m” is contradictory in one sentence. The first value should be the burial depth.

11. In Table 2, “Yield tension (Pa) 1420e6” is a stress, not a tension. In FLAC3D the cable yield tension is a force; with the cross-sectional area of 396e−6 m² it should be about 562 kN. The pretension and the exposed perimeter of the cable are also not given.

12. In Section 2.1, the FFA is said to be proposed by Yang in 2008, but Reference [20] is dated 2010. Reference [1] and several other entries are also not in the correct format, and References [12], [13], [18] and [19], which are about Schmidt hammer tests and DEM parameter calibration, are not related to roadway support. Please check the whole reference list.

13. In Section 2.3, “As shown in Fig., the starting point of a cable …” the figure number is missing. In Section 2.4, Figs. 5–8 are listed but Fig. 4 is omitted, and the caption of Fig. 1(b) is separated from the figure.

14. Some references should be added to enrich the research background. For example, Combined effects of fault geometry and roadway cross-section shape on the collapse behaviors of twin roadways: an experimental investigation, Failure mechanism and support technology of weakly cemented soft rock roadway in water-rich environment, Asymmetric large deformation in deep mining-damaged roadways: mechanism and collaborative control technology by means of destressing-bolting.

15. The English of the manuscript should be polished by a professional editor. Several sentences are repeated word by word, and some expressions are not standard.

**********

what does this mean?). If published, this will include your full peer review and any attached files.

If you choose “no”, your identity will remain anonymous but your review may still be made public.

**Do you want your identity to be public for this peer review?**  For information about this choice, including consent withdrawal, please see our Privacy Policy

Reviewer #1: No

Reviewer #2: No

**********

[NOTE: If reviewer comments were submitted as an attachment file, they will be attached to this email and accessible via the submission site. Please log into your account, locate the manuscript record, and check for the action link "View Attachments". If this link does not appear, there are no attachment files.]

To ensure your figures meet our technical requirements, please review our figure guidelines: https://journals.plos.org/plosone/s/figures

You may also use PLOS’s free figure tool, NAAS, to help you prepare publication quality figures: https://journals.plos.org/plosone/s/figures#loc-tools-for-figure-preparation.

NAAS will assess whether your figures meet our technical requirements by comparing each figure against our figure specifications.

# Author comment: Author response to Decision Letter 1

**DOI:** 10.1371/journal.pone.0357258.r002

10 Aug 2026

response to editor

1. Please upload a Response to Reviewers letter which should include a point by point response to each of the points made by the Editor and / or Reviewers. (This should be uploaded as a 'Response to Reviewers' file type.) Please follow this link for more information: http://blogs.PLOS.org/everyone/2011/05/10/how-to-submit-your-revised-manuscript/

Response: the response to reviewers letter include a point by point response to each points made by editor and reviewers has be uploaded.

2. It appears that your ORCiD iD has not been validated in your Editorial Manager account and we are unable to proceed until that step is complete.

To validate your ORCiD iD in Editorial Manager, please follow the steps below:

a. In your Editorial Manager account, please go to ‘Update my Information’ (in the upper left-hand corner of the main menu), and click on the Fetch/Validate link next to the ORCiD field.

b. This link will take you to the ORCiD site and allow you to create a new iD or authenticate a pre-existing iD in Editorial Manager.

For additional instructions, please watch the following video for a step-by-step demonstration: https://www.youtube.com/watch?v=_xcclfuvtxQ

Response: the ORCID has been validated.

3. Please provide a complete Data Availability Statement in the submission form, ensuring you include all necessary access information or a reason for why you are unable to make your data freely accessible. If your research concerns only data provided within your submission, please write "All data are in the manuscript and/or supporting information files" as your Data Availability Statement.

Response: data statement “All data are in the manuscript and/or supporting information files” was added.

4. It is important that you include a cover letter with your manuscript. Please ensure that this letter is addressed specifically to PLoS ONE. Please also include

* why this manuscript is suitable for publication in PLoS ONE.

* how does your paper provide a worthwhile addition to the scientific literature?

* how does your paper relate to previously published work?

* which types of scientists do you believe will be most interested in your study?

Response: the cover letter was revised.

5. Please amend the title either on the online submission form or in the manuscript so that they are identical.

Response: the title on the online submission form was revised.

6. Thank you for stating the following in your Competing Interests section:

“no”

Please complete your Competing Interests on the online submission form to state any Competing Interests. If you have no competing interests, please state "The authors have declared that no competing interests exist.", as detailed online in our guide for authors at http://journals.plos.org/plosone/s/submit-now

This information should be included in your cover letter; we will change the online submission form on your behalf.

Response: the competing interests was revised.

7. Thank you for stating the following in the Competing Interests section:

“no”

We note that one or more of the authors are employed by a commercial company: “China Coal Underground Space Technology (Shenzhen) Co., Ltd.”

1. Please provide an amended Funding Statement declaring this commercial affiliation, as well as a statement regarding the Role of Funders in your study. If the funding organization did not play a role in the study design, data collection and analysis, decision to publish, or preparation of the manuscript and only provided financial support in the form of authors' salaries and/or research materials, please review your statements relating to the author contributions, and ensure you have specifically and accurately indicated the role(s) that these authors had in your study. You can update author roles in the Author Contributions section of the online submission form.

Please also include the following statement within your amended Funding Statement.

“The funder provided support in the form of salaries for authors [insert relevant initials], but did not have any additional role in the study design, data collection and analysis, decision to publish, or preparation of the manuscript. The specific roles of these authors are articulated in the ‘author contributions’ section.”

If your commercial affiliation did play a role in your study, please state and explain this role within your updated Funding Statement.

Response: The commercial company was deleted.

Response to reviewers:

Reviewer #1: This study integrates firefly algorithm (FFA) with FLAC3D numerical simulation. Taking Banji Coal Mine roadway as the research object, FFA is adopted to optimize grouting range and anchor cable layout. Field application verifies that simulation results match practical data well. The study is meaningful and interesting, however, some questions and revision should be taken before the acceptance.

Response: Thanks for your comments, based on your comments, the page number and line number were added in the manuscript, meanwhile, the exact changes made in the revise manuscript, including corresponding page and line numbers were given.

(1)In this study, only one Coal Mine was taken as an example, the proposed method can be suitable for other coal mine

Response: Thanks for your comments, the proposed method can be applied to other coal mine, in the manuscript, once the shape of roadway and rock mechanic parameters were determined, the optimal grouting area and cable distribution can be determined based on the proposed method.

(2)The FFA algorithm was used to find the optimal parameters for water prevention, why this algorithm was selected? please explain the reasons.

Response: Thanks for your comments, many other optimization algorithm can be also used in the manuscript. In our manuscript, the FFA algorithm was selected, the FFA has several advantages: first, it features a simple and intuitive mechanism with few adjustable parameters, making it easy to understand, implement and program. Second, it exhibits strong global search capability, especially for multimodal optimization problems, as its attraction mechanism allows fireflies to automatically cluster into subgroups and effectively avoid premature convergence to local optima. Third, it achieves a good balance between global exploration and local exploitation by adjusting randomness and absorption coefficients. The detailed process of FFA algorithm is described below. The merits of the FFA algorithm was given in part 2.1 of the manuscript.

(3)In this paper, the FFA algorithm and FLAC3D numerical simulation combined by Python language, are you sure the Python can be used for FLAC3D numerical simulation?

Response: Thanks for your comments, the FLAC3D has two secondary development language, one is the FISH language and the other one is the Python script language, the Python language was supported since Version 5.0, which can be seen the manual of FLAC3D.

(4)The obtained numerical simulation results were applied to engineering practice, the difference between numerical simulation and engineering practice, how do you determine the mechanical parameters of rock mass in numerical simulation.

Response: Thanks for your comments, in our manuscript, the rock mass mechanical parameters were obtained from physical tests, and then the rock mass mechanical parameters were calibrated based on the rock mass grade. It should be noted that numerical simulation results agree well with engineering practice, which indicated that the rock mass mechanical parameters applied to numerical simulation model is reliable.

(5)How do you determine the range of cable length in the manuscript.

Response: Thanks for your comments, in our manuscript, the range of cable length is within the range of 0.1m and 10m, in fact, wider of the range cable length can be more capable to obtain the optimal cable length, to reduce the iteration time, hence, the range (0.1, 10) was selected.

This paper can be accepted after minor revision.

Response: Thank you again. There is no questions needed to be answered.

Reviewer #2: The manuscript titled “Optimal Parameters of Roadway Supports for Water Prevention based on the FFA Algorithm” combines the firefly algorithm (FFA) with FLAC3D fluid–mechanical simulation to determine the optimal grouting area and cable layout of a water-bearing roadway, and the optimized scheme was applied to the Banji Coal Mine. The topic is of practical value in mining engineering, and the field implementation is a strength that many optimization studies do not have. However, several material parameters and boundary conditions are physically inadmissible or contradictory, and most of the information needed to reproduce the work is missing, so the results cannot be assessed in the current version. Please see my comments below:

Response: Thanks for your comments, based on your comments, the page number and line number were added in the manuscript, meanwhile, the exact changes made in the revise manuscript, including corresponding page and line numbers were given.

1. In Table 1, the bulk modulus (3.1 GPa) is much smaller than the shear modulus (9.5 GPa), which gives a negative Poisson’s ratio for the rock mass (ν = −0.26). The grouting area gives ν = −0.24. This is not possible for coal measure rock. If the two columns are exchanged, ν ≈ 0.35, which is reasonable. Please check the laboratory data, correct Table 1 and re-run all the simulations, because every mechanical result depends on these values.

Response: Thanks for your comments, it is our mistakes, the two columns should be exchanged, our numerical simulation are correct, there exist some mistakes when writing the manuscript, there is no need to re-run all the simulations. The changes were highlighted by red color, which can be seen in the revised manuscript.

2. The seepage boundary condition is contradictory. The text says the model boundary is a waterproof boundary, the water pressure at the roadway edge is 0 MPa, and the flow is steady, but Fig. 3 shows a water pressure of 2.3 MPa applied at the bottom over 8 m. If all outer boundaries are impermeable and the roadway is a drain, the steady-state inflow should be zero, and the reported 0.169 m³/h cannot be obtained. Please give the complete hydraulic boundary conditions and make the text consistent with Fig. 3.

Response: Thanks for your comments, based on your comments, the model boundary of the numerical simulation was changed. In our manuscript, the roadway surrounding rock surface is set as zero pore water pressure drainage boundary. A constant water pressure of 2.3 MPa is applied to the bottom aquifer within 8 m height to simulate deep confined water recharge. The top, left and right boundaries of the numerical model are all defined as impermeable no-flow boundaries. Groundwater flows steadily from the high-pressure bottom water-rich zone to the roadway drainage surface, and the stable water inflow is finally calculated. The changes were highlighted by red color, which can be seen in the revised manuscript.

3. The dimension of the optimization problem is not consistent. Section 2.3 lists ten grouting lengths, the cable number, the cable length, and the starting point and inclination angle of each cable, which is at least 34 variables for 11 cables, but Step 1 of the same section says the variable is ten-dimensional. Please give the variable vector, its dimension, and the search range of each variable in a table.

Response: Thanks for your comments, based on your comments, the initialized parameters and ranges were given in Table 1, the changes were highlighted by red color, and the changed can be seen in the revised manuscript.

4. The parameters of the FFA are not reported. The population size, α, β₀, γ and max_iter actually used are not given, only the general ranges in Section 2.1, and there is no convergence curve, iteration number, total number of FLAC3D calls, or computation time. As FFA is a stochastic algorithm, please add the hyperparameters, the convergence history, and several independent runs with the mean and scatter of the best objective value.

Response: Thanks for your comments, based on your comments, in this paper, for the FFA algorithm, the parameters were set as follows: Initialize the parameters of the firefly algorithm (FFA), in this paper, the population size is 50, randomization parameter is 0.02, initial attractiveness at distance 0 is 1.0, the light absorption coefficient is 2.0, the maximum number of iterations is 1000, the changes were added in the revised manuscript. Meanwhile, based on your comments, the convergence history, and the mean and scatter of the best objective value was given in the manuscript.

5. The numerical model is not described. Please add the model dimensions, element size, number of zones, and a mesh sensitivity analysis. The porosity, Biot coefficient and fluid bulk modulus required by Equations (10)–(13) are not given, and the unit of the permeability coefficient (7.9e−11 and 4.5e−13) is missing, so these values cannot be interpreted.

Response: Thanks for your comments, based on your comments, the numerical simulation model is described, the model dimensions, element size, number of zones were given, meanwhile, the porosity, Biot coefficient and fluid bulk modulus are given, which can be seen in the revised manuscript.

6.In Section 2.3, Step 3, the objective value of an infeasible solution is set to infinity. If most of the initial population is infeasible, all fireflies have the same brightness, the attraction term disappears, and the algorithm degenerates into a random search. Please report the feasible proportion of the initial population, and consider a graded penalty function or a repair operator.

Response: Thanks for your comments, if the condition is not satisfied, the objective value shall be assigned the value calculated by Equation (16) plus 1e7.

7. Section 2.2 repeats the FLAC3D theory manual (Reference [21]) at too much length. Equations (6)–(13) are presented but the parameters they introduce (α, φ, K_f, M_b) are never assigned values or used afterwards. Please shorten this section and keep only the formulation and parameters actually adopted, with the source clearly cited.

Response: Thanks for your comments, based on your comments, this part was shorten, which can be seen in the revised manuscript.

8. The grouting area is described by ten rays at 36° interval, but the reason for choosing ten is not explained and no sensitivity analysis of the number of control points is given. The optimized boundary in Table 3 (4.90–7.98 m) is close to an ellipse, so it is not clear how much the ten parameters contribute. Please also discuss whether such a shape can actually be constructed by borehole grouting.

Response: Thanks for your comments, as a matter of fact, more accuracy of the numerical results would be obtained if more end points selected. If more end points were used, then more variables needed to be determined during the process, in our manuscript, ten end points were determined based on the experience, meanwhile, the end points were connected by cubic spline interpolation method, the closed line is smooth, and the results were acceptable. However, the actual grouting depth of roadways after grouting can be comprehensively determined by construction parameters, on-site verification and non-destructive testing. The slurry diffusion range is preliminarily judged according to the final drilling depth, changes of grouting pressure and flow rate, and slurry consumption. After the slurry solidifies, core sampling and borehole inspection are conducted through inspection holes arranged between grouting holes to directly detect the maximum effective filling depth of slurry veins. Meanwhile, geophysical methods such as ground penetrating radar and acoustic wave detection are adopted to define the grouting reinforcement boundary, and the actual effective grouting depth of roadways is finally confirmed by mutual verification of various data. I hope my answer can meet your satisfactory.

9. The water inflow decreases from 97 m³/h to 0.13 m³/h, about 750 times, while the permeability ratio of the rock mass and the grouting area is only about 176. Please give a simple consistency check of this reduction against the grouting geometry.

Response: Thanks for your comments, The discrepancy is mainly caused by the change of seepage flow channel geometry after grouting. Grouting not only reduces rock mass permeability, but also greatly narrows, blocks and disconnects original dominant seepage paths. The actual seepage cross-sectional area decreases sharply, leading to a far larger multiple of water inflow reduction than the permeability ratio, which is consistent with the field grouting geometric characteristics. Meanwhile, the numerical simulation was repeated, the numerical simulation results were right.

10. In Section 2.4, “The roadway height was less than 636 m, the height of the roadway was 3.2 m” is contradictory in one sentence. The first value should be the burial depth.

Response: Thanks for your comments, the contradictory part was revised, which can be seen in the revised manuscript.

11. In Table 2, “Yield tension (Pa) 1420e6” is a stress, not a tension. In FLAC3D the cable yield tension is a force; with the cross-sectional area of 396e−6 m² it should be about 562 kN. The pretension and the exposed perimeter of the cable are also not given.

Response: Thanks for your comments, the unit of yield tension is revised, the pretension and the exposed perimeter of the cable are added in the manuscript.

12. In Section 2.1, the FFA is said to be proposed by Yang in 2008, but Reference [20] is dated 2010. Reference [1] and several other entries are also not in the correct format, and References [12], [13], [18] and [19], which are about Schmidt hammer tests and DEM parameter calibration, are not related to roadway support. Please check the whole reference list.

Response: Thanks for your comments, FFA is said to be proposed by Yang in 2010, which can be seen in the revised manuscript, and references [12], [13], [18] and [19] were deleted, and whole reference list were checked.

13. In Section 2.3, “As shown in Fig., the starting point of a cable …” the figure number is missing. In Section 2.4, Figs. 5–8 are listed but Fig. 4 is omitted, and the caption of Fig. 1(b) is separated from the figure.

Response: Thanks for your comments, in section 2.3, the figure number is added. Meanwhile, the Fig.4 is added. The Fig. 1 in the manuscript was revised.

14. Some references should be added to enrich the research background. For example, Combined effects of fault geometry and roadway cross-section shape on the collapse behaviors of twin roadways: an experimental investigation, Failure mechanism and support technology of weakly cemented soft rock roadway in water-rich environment, Asymmetric large deformation in deep mining-damaged roadways: mechanism and collaborative control technology by means of destressing-bolting.

Response: Thanks for your comments, based on your comments, some references were added to enrich the research background.

15. The English of the manuscript should be polished by a professional editor. Several sentences are repeated word by word, and some expressions are not standard.

Response: Thanks for your comments, based on your comments, our manuscript was polished by American Journal Expert, the editing proof was attached. The changed part was highlighted by red color, which can be seen in the revised manuscript.

Attachment Submitted filename: Response to reviewers.doc

# Peer review: Decision Letter 1

**DOI:** 10.1371/journal.pone.0357258.r003

**Authors:** Zhenhua Li

16 Aug 2026

Optimal Parameters of Roadway Supports for Water Prevention based on the FFA Algorithm

PONE-D-26-35180R1

Dear Dr. xu,

We’re pleased to inform you that your manuscript has been judged scientifically suitable for publication and will be formally accepted for publication once it meets all outstanding technical requirements.

Within one week, you’ll receive an e-mail detailing the required amendments. When these have been addressed, you’ll receive a formal acceptance letter and your manuscript will be scheduled for publication.

An invoice will be generated when your article is formally accepted. Please note, if your institution has a publishing partnership with PLOS and your article meets the relevant criteria, all or part of your publication costs will be covered. Please make sure your user information is up-to-date by logging into Editorial Manager at Editorial Manager® and clicking the ‘Update My Information' link at the top of the page. For questions related to billing, please contact billing support.

If your institution or institutions have a press office, please notify them about your upcoming paper to help maximize its impact. If they’ll be preparing press materials, please inform our press team as soon as possible -- no later than 48 hours after receiving the formal acceptance. Your manuscript will remain under strict press embargo until 2 pm Eastern Time on the date of publication. For more information, please contact onepress@plos.org.

Kind regards,

Zhenhua Li

Academic Editor

PLOS One

Additional Editor Comments (optional):

accept

Reviewers' comments:

Reviewer's Responses to Questions

**Comments to the Author**

Reviewer #1: All comments have been addressed

Reviewer #2: All comments have been addressed

**********

2. Is the manuscript technically sound, and do the data support the conclusions?

Reviewer #1: Yes

Reviewer #2: Yes

**********

3. Has the statistical analysis been performed appropriately and rigorously?

Reviewer #1: Yes

Reviewer #2: Yes

**********

4. Have the authors made all data underlying the findings in their manuscript fully available?

The PLOS Data policy

Reviewer #1: Yes

Reviewer #2: Yes

**********

5. Is the manuscript presented in an intelligible fashion and written in standard English?

Reviewer #1: Yes

Reviewer #2: Yes

**********

Reviewer #1: All reviewer comments have been satisfactorily resolved. The manuscript is now ready for acceptance.

Reviewer #2: I have no further comments on this manuscript, and this manuscript can be accepted for publication after check the grammar issues.

**********

what does this mean?). If published, this will include your full peer review and any attached files.

If you choose “no”, your identity will remain anonymous but your review may still be made public.

**Do you want your identity to be public for this peer review?**  For information about this choice, including consent withdrawal, please see our Privacy Policy

Reviewer #1: **Yes:**  yes

Reviewer #2: No

**********

# Editor report: Acceptance letter

**DOI:** 10.1371/journal.pone.0357258.r004

**Authors:** Zhenhua Li

PONE-D-26-35180R1

PLOS One

Dear Dr. xu,

I'm pleased to inform you that your manuscript has been deemed suitable for publication in PLOS One. Congratulations! Your manuscript is now being handed over to our production team.

At this stage, our production department will prepare your paper for publication. This includes ensuring the following:

* All references, tables, and figures are properly cited

* All relevant supporting information is included in the manuscript submission,

* There are no issues that prevent the paper from being properly typeset

You will receive further instructions from the production team, including instructions on how to review your proof when it is ready. Please keep in mind that we are working through a large volume of accepted articles, so please give us a few days to review your paper and let you know the next and final steps.

Lastly, if your institution or institutions have a press office, please let them know about your upcoming paper now to help maximize its impact. If they'll be preparing press materials, please inform our press team within the next 48 hours. Your manuscript will remain under strict press embargo until 2 pm Eastern Time on the date of publication. For more information, please contact onepress@plos.org.

You will receive an invoice from PLOS for your publication fee after your manuscript has reached the completed accept phase. If you receive an email requesting payment before acceptance or for any other service, this may be a phishing scheme. Learn how to identify phishing emails and protect your accounts at https://explore.plos.org/phishing.

If we can help with anything else, please email us at customercare@plos.org.

Thank you for submitting your work to PLOS One and supporting open access.

Kind regards,

PLOS One Editorial Office Staff

on behalf of

Professor Zhenhua Li

Academic Editor

PLOS One
